# The Oscillatory Profile Induced by the Anxiogenic Drug FG-7142 in the Amygdala–Hippocampal Network Is Reversed by Infralimbic Deep Brain Stimulation: Relevance for Mood Disorders

**DOI:** 10.3390/biomedicines9070783

**Published:** 2021-07-06

**Authors:** Hanna Vila-Merkle, Alicia González-Martínez, Rut Campos-Jiménez, Joana Martínez-Ricós, Vicent Teruel-Martí, Arantxa Blasco-Serra, Ana Lloret, Pau Celada, Ana Cervera-Ferri

**Affiliations:** 1 Neuronal Circuits Laboratory, Department of Human Anatomy and Embryology, University of Valencia, 46010 Valencia, Spain; hanna.vila@uv.es (H.V.-M.); gonmara3@alumni.uv.es (A.G.-M.); rutcj97@gmail.com (R.C.-J.); vicent.teruel@uv.es (V.T.-M.); 2 GESADA Laboratory, Department of Human Anatomy and Embryology, University of Valencia, 46010 Valencia, Spain; arantxa.blasco@uv.es; 3 Department of Physiology, Faculty of Medicine, University of Valencia, 46010 Valencia, Spain; ana.lloret@uv.es; 4 Health Research Institute INCLIVA, 46010 Valencia, Spain; 5 Department of Neurochemistry and Neuropharmacology, CSIC-Institut d’Investigacions Biomèdiques de Barcelona (IIBB-CSIC), 08036 Barcelona, Spain; pau.celada@iibb.csic.es; 6 Institut d’Investigacions Biomèdiques August Pi i Sunyer (IDIBAPS), 08036 Barcelona, Spain; 7 Centro de Investigación Biomédica en Red de Salud Mental (CIBERSAM), 08036 Barcelona, Spain

**Keywords:** oscillations, anxiety, deep brain stimulation, electrophysiology, prefrontal, hippocampus, amygdala

## Abstract

Anxiety and depression exhibit high comorbidity and share the alteration of the amygdala–hippocampal–prefrontal network, playing different roles in the ventral and dorsal hippocampi. Deep brain stimulation of the infralimbic cortex in rodents or the human equivalent—the subgenual cingulate cortex—constitutes a fast antidepressant treatment. The aim of this work was: (1) to describe the oscillatory profile in a rodent model of anxiety, and (2) to deepen the therapeutic basis of infralimbic deep brain stimulation in mood disorders. First, the anxiogenic drug FG-7142 was administered to anaesthetized rats to characterize neural oscillations within the amygdala and the dorsoventral axis of the hippocampus. Next, deep brain stimulation was applied. FG-7142 administration drastically reduced the slow waves, increasing delta, low theta, and beta oscillations in the network. Moreover, FG-7142 altered communication in these bands in selective subnetworks. Deep brain stimulation of the infralimbic cortex reversed most of these FG-7142 effects. Cross-frequency coupling was also inversely modified by FG-7142 and by deep brain stimulation. Our study demonstrates that the hyperactivated amygdala–hippocampal network associated with the anxiogenic drug exhibits an oscillatory fingerprint. The study contributes to comprehending the neurobiological basis of anxiety and the effects of infralimbic deep brain stimulation.

## 1. Introduction

The present paper analyzes the effects of an anxiogenic drug, *N*-methyl-β-carboline-3-carboxamide (FG-7142), on the oscillatory activity of the prefrontal–amygdala–hippocampal network, which is altered in both anxiety and depression. Furthermore, this study explores whether anxiogenic-induced changes in the network can be reversed by deep brain stimulation (DBS) of the infralimbic cortex (IL-DBS) in urethane-anaesthetized rats. With this preclinical study, we aimed to contribute to the understanding of the neurobiological basis of anxiety, and to assess whether the IL-DBS could modify an aberrant connectivity in this network.

### 1.1. Depression and Anxiety as Comorbid Disorders

Depression and anxiety are among the most prevalent mental disorders considered “common” mental disorders, and they could be classified as part of an emergent pandemic of mood disorders [1]. According to the World Health Organization’s (WHO) Global Health Estimates [2], more than 320 million people worldwide suffer from depression, with a growing global prevalence to 4.4% in 2015. Regarding its prevalence, anxiety is the second most common mental disorder, affecting 264 million people globally (3.6%). Both disorders are among the most significant contributors to non-fatal health loss, measured as years lived with a disability. In addition, mental disorders are stronger predictors of suicidal behavior, which is a leading cause of death worldwide, causing almost 800,000 deaths every year. While major depressive disorder is the strongest predictor of suicidal ideation, comorbidity with anxiety increases the possibility of suicide plans or attempts [3,4]. 

According to the Diagnostic and Statistical Manual of the American Psychiatric Association (DSM-V), major depressive disorder (MDD) is a syndrome that includes episodes of persistent negative mood or anhedonia, together with additional emotional, psychological, and somatic symptoms [5]. To be considered MDD, these symptoms must persist for at least two weeks and not be secondary to any substance, medical condition, or other psychiatric disorder; nor can the symptoms be better explained by normal grief. On the other hand, clinical anxiety involves a maladaptative “marked, persistent, and excessive or unreasonable fear”, which significantly interferes with everyday life. 

Anxiety and depression are often comorbid and, when they occur together, the pathology presents a diminished clinical outcome and quality of life [6]. Indeed, according to epidemiological data of The Netherlands Study of Depression and Anxiety [7], 67% of individuals suffering from depressive disorder had a current anxiety disorder and 75% had a lifetime comorbid anxiety disorder. On the other hand, 63% of patients with a current anxiety disorder had a simultaneous depressive disorder, and 81% had a lifetime depressive disorder. Usually, anxiety symptoms precede depression. Additionally, patients presenting comorbidity of both pathologies present higher symptom severity, which is the prognosis for comorbid anxiety and depression that is worse than either condition alone. Thus, there is an alternative proposal for a continuum model for anxiety syndromes including mild, moderate, and severe/psychotic depression [1,8]. 

A significant problem is finding fast-acting treatments that reverse aberrant neural activity. Despite the high incidence and relevance of depression, to date, antidepressant therapies are not helpful for all patients, and one-third of people suffering MDD are not responders [9]. The failure of usual antidepressant treatments may be due to a lack of understanding of the precise neurobiological basis of MDD and the constellations of symptoms included in the syndrome [10]. According to Holtzheimer and Mayberg, the goal of antidepressant therapy should be to maintain normal mood regulation over time instead of an acute resolution of the symptoms.

### 1.2. Prefrontal Deep Brain Stimulation in the Treatment of Depression

However, DBS in different targets has generated promising results in various mental disorders. Its success resides in manipulating the precise malfunctioning circuits in a broad range of disorders, including psychiatric conditions [11,12]. Thus, it is a different therapeutic approach and a powerful tool to study altered brain networks. Nevertheless, the underlying mechanisms require further research. 

In depression, DBS has proven to induce immediate positive subjective experiences in patients [13], with sustained effects on mood, anxiety, sleep, and somatic symptoms after months of stimulation [13,14,15,16]. Additionally, following DBS, a reduction in anxiety in seconds to minutes, followed by mood changes in timing from hours to days, has been reported [17,18]. Since 2005, different targets have been used in clinical studies for treatment-resistant depression, with electrodes targeting regions altered in the pathology, such as the subcallosal or subgenual anterior cingulate cortex (sACC), the equivalent Brodmann area 25/24 [13,15,19,20], ventral striatum [19,20], and medial forebrain bundle [21]. 

Regional cerebral blood flow in sACC increases in depression [22] and anticipatory anxiety [23], and is related to negative affect in healthy subjects [24]. Based on this hyperactivity in negative affect, Mayberg and colleagues targeted sACC for DBS for the first time in MDD patients [13]. In this seminal work, the patients showed acute and short-term beneficial effects of the DBS, time-locked with the stimulation and without effects elicited by sham stimulation. Together with the antidepressant response, the regional cerebral blood flow in the sACC decreased. Puigdemont et al. also reported an early response, with a high response and remission rate at 1 week and lasting effects, with a remission rate of 50% after 1 year of treatment [25]. According to the meta-analysis conducted by Zhou et al., sACC DBS significantly alleviates depressive symptoms [26]. Khairuddin and colleagues [27] wrote a recent review of subcallosal DBS for treating depression that reported long-lasting maintenance of an antidepressant response, reducing the occurrence of recurrent depressive episodes; this treatment was usually well tolerated. From 39 clinical studies, they found that response rate increased with treatment duration from 63.8% at ≤6 months to 76% at ≥24 months, and a remission rate ranging from 36.5% to 62.5%. Recently, an 8-year follow-up study from Mayberg’s group with 28 patients reported response and remission rates of 50% and 30%, respectively. However, there is not a conclusive consensus regarding whether DBS for depression, and likely for anxiety, is effective, since a multisite, randomized, sham-controlled trial also detected positive effects in sham-operated patients [28]. The authors suggested that these controversial effects could be due to a placebo effect, clinical features of the patient population, or suboptimal electrode placement. Nevertheless, systematic reviews and meta-analyses following standard protocols [29], such as PRISMA (Preferred Reporting Items for Systematic Reviews and Meta-analyses), on DBS effects are needed to confirm its efficacy and optimize this surgical intervention. Additionally, further clinical and preclinical studies are needed to gain a better understanding of DBS mechanisms. 

Given the involvement of the prefrontal cortex in psychiatric disorders, analyzing its activity in animal models is of interest. However, the homology between human and rodent prefrontal cortex [30], or even the existence of prefrontal cortex in non-primate mammals [31,32], is controversial. The divergence primarily refers to the absence of a granular layer and the lack of exact rodent correlation to the dorsolateral prefrontal cortex. However, comparing cortical areas of distant species based on cytoarchitectonic criteria has been questioned [31], and there is evidence of a rodent behavioral and connectional homology of the medial prefrontal cortex (mPFC) [30]. The rat mPFC can be subdivided into a dorsal and a ventral component based on several anatomical and functional criteria. The latter includes the ventral prelimbic, infralimbic (IL), and medial orbital areas [33].

A recent study compared rat, marmoset, and human brain functional connectivity in the resting-state by functional magnetic resonance imaging (fMRI). The authors found that area 25 was most similar in all three species, unlike other medial prefrontal regions, which showed a higher divergence between rats and primates [34]. Indeed, they found robust functional connectivity between area 25 and amygdala in rats and primates, which is relevant for its involvement in mood disorders. In the rodent brain, area 25 corresponds to the IL region of the mPFC [34,35,36] Tracing studies also report similar connectivity between the IL and the sACC, with the most robust connections found for the hypothalamus, nucleus accumbens, the fornix, and the medial temporal lobe, including the amygdala, insula, and anterior hippocampus in human, or the ventral hippocampus [35,36], which is the equivalent in a rodent brain [37]. Moreover, the IL region is susceptible to stressful or anxiogenic stimuli, and its lesioning induces anxiolytic responses in the elevated plus-maze test [34] and decreases plasma corticosterone levels in response to acute restraint [38].

Additionally, in rats, IL-DBS has fast antidepressant-like and anxiolytic-like effects [39,40,41]: the use of 1 h of IL-DBS reduces immobility time and increases climbing behavior in the forced swimming test; it also reduces the latency in feeding in the novelty-suppressed feeding test. Neurochemically, IL-DBS delivered for one hour increases the release of glutamate, serotonin, dopamine, and noradrenaline in the mPFC [40,41]. Although DBS’s neurochemical and behavioral effects have been examined, less attention has been paid to the influence of DBS on the network dynamics between different brain areas. To date, no previous study has investigated the effects of IL-DBS in a pharmacological model of anxiety. Thus, research on neural systems involved in anxiety and depression could improve our understanding of mood disorders’ neurobiology and help design better targets for intervention.

### 1.3. The Amygdala–Hippocampal–Prefrontal Network in Depression and Anxiety 

Mood disorders present an alteration in the amygdala–hippocampal–prefrontal network. Within the control network of emotional processing, the mPFC plays a key role. Dysfunction of this circuitry is one of the hallmarks of depression and anxiety [42].

In humans, imaging studies have displayed a decreased hippocampal volume in clinical anxiety [43] and depression [44,45], as well as in hippocampal microstructure abnormalities in comorbid anxiety and depression, which are associated with exacerbated threat processing [46]. Rodent studies have also evidenced alterations in the hippocampus in clinical anxiety [47], with a stronger association with anxiety than depression-like behavior [48]. In addition, hypersecretion of glucocorticoids involved in the acute and chronic stress response induces alterations in neuroplasticity in the hippocampus, leading to atrophy and neuronal loss in depression [49,50]. However, the involvement of the hippocampus in mood disorders is not fully understood. 

The hippocampus is anatomically, neurochemically, and functionally subdivided along the septotemporal axis into dorsal, intermediate, and ventral regions [51,52,53,54]. While the dorsal hippocampus (dHPC) is mainly related to cortical regions and involved in cognitive processing, the ventral hippocampus (vHPC) has reciprocal projections to the amygdala and hypothalamus, and processes emotional and stress-related behaviors. However, Strange and colleagues suggested a gradient of functional hippocampal domains instead of precise distributed functions [37]. In addition, the neurochemical organization of the hippocampal formation evidences a high density of adrenergic receptors in the ventral region [54], which is relevant in stress response and depression and their links with decreased neurogenesis. 

On the other hand, the amygdala is pivotal in normal emotional processing [55,56] and changes in its activity are related to anxiety and depression. In rodents, chronic stress induces hyperexcitability [57] and maladaptive stress leading to anxiety induces plastic changes in the amygdala [58,59]. An over-reactive amygdala has also been evidenced both in anxiety-prone humans [60] and in depressed patients by imaging studies [61]. Antidepressants can reverse this altered activity, as can gene expression [62,63,64]. Finally, lesion studies highlight that the amygdala plays a significant role not only in the establishment of depression-like behaviors but also in hippocampal atrophy [65]. Under normal conditions, the stress response of the amygdala is under control of the ventromedial prefrontal cortex, while the disruption of the prefrontal control is found in mental illnesses [66].

In a previous study, we demonstrated that IL-DBS improves communication and functional connectivity in the amygdala–hippocampal network in normal anaesthetized rats [67]. However, its effect on anxious animals has not yet been analyzed. Modelling psychiatric disorders is not a straightforward question and a spectrum of experimental models of anxiety is available; however, these models’ validity has faced criticism [68,69]. In the present paper, we have chosen a proven anxiogenic drug to study the amygdala–hippocampal network and its modulation by IL-DBS.

### 1.4. FG-7142 as an Anxiogenic Drug

β-carbolines are stress-sensitive compounds present in the rat brain that are excreted in urine and act as benzodiazepine inhibitors [70]. The β-carboline FG-7142 acts as a partial inverse agonist at the benzodiazepine allosteric site of the GABAA receptor, with the highest affinity for the α1-subunit [71]. 

There is a growing body of evidence that this drug elicits anxious behaviors, both in freely moving rodents [72,73,74] and in humans [71], as well as activating neural networks underlying the anxious response [75]. Studies over the past five decades have provided information about the anxiogenic-like effects of FG-7142 in a variety of species, including mice, rats, cats, monkeys, and humans. Experimental testing in humans reported severe anxiogenic and panic-like responses following administration of β-carboline, which has been the reason for stopping human research with the drug [76,77]. Given the large body of literature reports on the behavioral effects of FG-7142 as a pharmacological model of anxiety, our study was conducted under anesthesia to exclude other confounding factors, i.e., motor activity. However, we are aware that the lack of behavioral correlates and the anesthesia itself could be considered limitations, as we will discuss further. 

Evans and Lowry performed a detailed compilation of FG-7142 effects in many experimental paradigms [71]. In rats, FG-7142 induces an increase of nonambulatory motor activity, which is related to increased arousal and vigilance [78]. Additionally, its administration exerts a proconflicting action, enhances shock-induced drinking suppression [79], reduces exploratory behavior in both mice and rats [80,81], enhances behavioral suppression in response to safety signal withdrawal [82], and impairs fear extinction [83]. In the elevated plus-maze, many rodent studies found that FG-7142 reduces the exploration time spent in the open arms and increases the time spent in the closed arms [71]. Several authors have also described a reduction in social interaction in rodents [84,85]. 

In accordance with its behavioral effects, FG-7142 increases corticosterone in the plasma levels of rats [86,87]. Additionally, there are reports, both in monkey and in cats, of FG-7142 actions on behavioral agitation and autonomic responses, including increases in heart rate and blood pressure and rises in plasma cortisol, corticosterone, and catecholamines [88,89]. In cats, FG-7142 also generates a state of elevated arousal and fearfulness [90]. In rats and mice, at similar doses, the drug increases c-fos expression in a widespread network involved in stress, anxiety, and fear-related behavioral and autonomic responses. This network includes forebrain structures such as the amygdala, ventral hippocampus, bed nucleus of the stria terminalis, cingulate, prelimbic and IL cortices, and brainstem nuclei of the reticular ascending arousal system, including locus coeruleus, periaqueductal grey, nucleus incertus, and dorsal raphe nucleus [74,75,78,91,92,93]. In addition, noradrenergic neurons in the locus coeruleus and serotonergic neurons in the dorsal raphe nucleus [74] are activated by FG-7142.

### 1.5. Brain Oscillations as a Measure of Network Dynamics

Network dynamics and information coding are reflected in brain oscillations [94]. These neural signals reflect the activity of distributed populations of neurons and can be recorded by means of surface electrodes aimed at the skull (electroencephalogram, EEG signals) or at the brain surface (electrocorticogram), or as local field potentials (LFPs) using intracranial electrodes inserted directly in the recorded area. LFPs emerge as the result of the interaction of local electrical activity (i.e., neuronal discharges) with postsynaptic potentials generated by the inputs from interconnected brain areas. Given their biophysical properties, there is an inverse relationship between the frequency of the oscillations and the extent of the network involved: while fast oscillations are influenced more by local neuronal activity, lower frequencies allow for communication among more distant areas [95]. Additionally, an increase in synchronization is usually assumed to reflect an increase in communication in the network [96]. Therefore, measuring oscillatory changes and isolating the relevant signals are useful for inferring the function of neural networks in normal conditions and mental disorders [97,98]. 

In this paper, we first analyze the oscillatory activity of the amygdala–hippocampal network in anaesthetized rats in basal conditions and after FG-7142 administration. LFPs were recorded in the dorsal, intermediate, and ventral subregions of the hippocampus and the basolateral amygdala. Next, we applied IL-DBS on the same animals to deepen the knowledge of the mechanisms of this therapeutic approach, focusing on its effects on the modulation of the amygdala–hippocampal network. 

In brief, our work showed that the anxiogenic drug FG-7142 induces a characteristic oscillatory profile in the amygdala–hippocampal circuit. Furthermore, 1 h of IL-DBS reverses most of the altered oscillations induced by FG-7142. Thus, the study suggests possible anxiety-related biomarkers and helps us understand the effects of IL-DBS on mood disorders. 

## 2. Materials and Methods

### 2.1. Animal Model and Surgical Procedures

To minimize the number of animals used and their suffering, behavioral experiments to evidence the anxious state were not replicated. However, we used FG-7142 doses, which have been extensively evidenced in the bibliography to induce anxious responses in rats [71], as well as an IL-DBS protocol that has already proved to have antidepressant and anxiolytic-like effects [40]. A total number of 24 male Wistar rats (250–350 g; Charles River Company, Barcelona, Spain) were used. Animals were housed in the Central Research Unit at the University of Valencia (Spain) under a 12 h/12 h light–dark cycle and a controlled temperature of 22 ± 2 °C and humidity (55 ± 10%). Food and water were available ad libitum. All the experimental protocols were followed according to the Animal Care Guidelines of the European Communities Council Directive (2010/63/E.U.) and approved by the Ethics Committee of the University of Valencia before performing the experiments.

**Figure 1:**
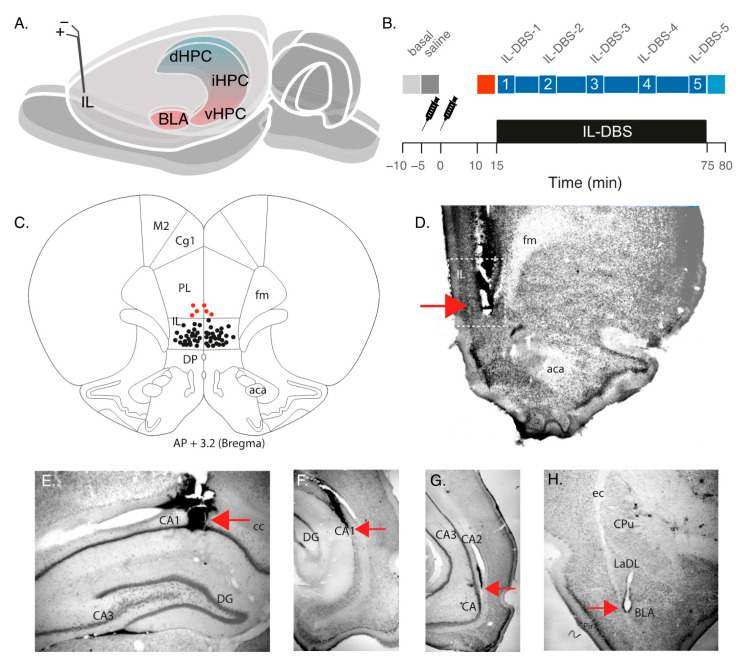
(**A**) Experimental setup. Top: recording and stimulating regions. Bottom: recording protocol with periods selected for statistical analysis. (**B**) Schematic diagram showing DBS electrode tip positions. Red dots indicate the electrode tips in the excluded exemplars. All stimuli were delivered bilaterally. (**C**) Histological sample showing the electrode track and tip in IL. (**D**) Electrode track and tip (red arrow) in dHPC. (**E**) Electrode track and tip (red arrow) in iHPC. (**F**) Electrode track and tip (red arrow) in vHPC. (**G**) Electrode track and tip (red arrow) in BLA. (**H**) Electrode track and tip (red arrow) in IL. aca: anterior commissure; BLA: basolateral amygdala; CA1, CA2, CA3: Cornu Ammonis fields; cc: corpus callosum; CPu: caudate putamen nucleus; DG: dentate gyrus; dHPC: dorsal hippocampus; fm: forceps minor; iHPC: intermediate hippocampus; IL: infralimbic cortex; LaDL: lateral amygdaloid nucleus, dorsolateral part; vHPC: ventral hippocampus.

Rats were anaesthetized with an intraperitoneal injection of 1.5 g/kg of urethane (Sigma-Aldrich/Merk, Barcelona, Spain) with supplemental doses to maintain the anesthetic level, complemented by local anesthesia with lidocaine sc (1 mL, 5%), blocking the zygomatic and ophthalmic nerves, and fibers. After the loss of corneal reflex and paw withdrawal, animals were placed inside a stereotaxic frame. Trephine holes were drilled in the skull and 100 µm-diameter cylindric, custom-made Teflon-coated stainless steel recording electrodes (AM Systems, Sequim, WA, USA) were placed, according to the stereotactic coordinates from Bregma [99]; these were always placed ipsilaterally in the left hemisphere at the dHPC (AP −3.4 mm; L 2.5 mm; DV 2.4 mm), intermediate hippocampus (iHPC) (AP −5.8 mm; L 5.8 mm; DV 5 mm), and vHPC (AP −4.7 mm; L 5 mm; DV 8.7 mm and basolateral amygdala (BLA) (AP −2.3 mm; L 5 mm; DV 8.5 mm). A stainless-steel screw (Plastics One, Roanoke, VA, USA) was implanted in the occipital bone as a reference, and was fixed with dental acrylic. For DBS, we used in-house custom-made bipolar twisted electrodes made of 100 µm-diameter Teflon-coated stainless steel wire, with 1 mm between both tips. Stimulating electrodes were bilaterally implanted into the IL region of the mPFC (AP +3.2 mm; L 0.5 mm; DV 5.4 mm). A correction factor was applied to the coordinates to increase accuracy, based on the ratio between the experimental and the theoretical distance from bregma to interaural references (experimental distance/9 mm). Additionally, different penetration angles were used to reach the desired coordinates (details in Supplementary Table S1). Recorded and stimulated regions are illustrated in Figure 1A,B. Body temperature was maintained throughout the operation with an isothermal pad at 37 °C. 

### 2.2. Drug Administration

As a control, before the administration of the anxiogenic drug, the animals were injected with 2 mL/kg saline solution ip FG-7142 (Sigma-Aldrich, St. Louis, MO, USA), which was administered ip at a single dose of 7.5 mg/kg diluted in 12.5 mL of β-cyclodextrin and 1 M chlorohydric acid (HCl), pH 5.0, to a final volume of 2 mL/kg [74]. 

Under urethane, FG-7142 induces the release of acetylcholine in the vHPC [100], an effect that was recently found under chronic restraint stress, which is a model of anxiety [101].

### 2.3. Recording and Stimulation Procedure

**Figure 2:**
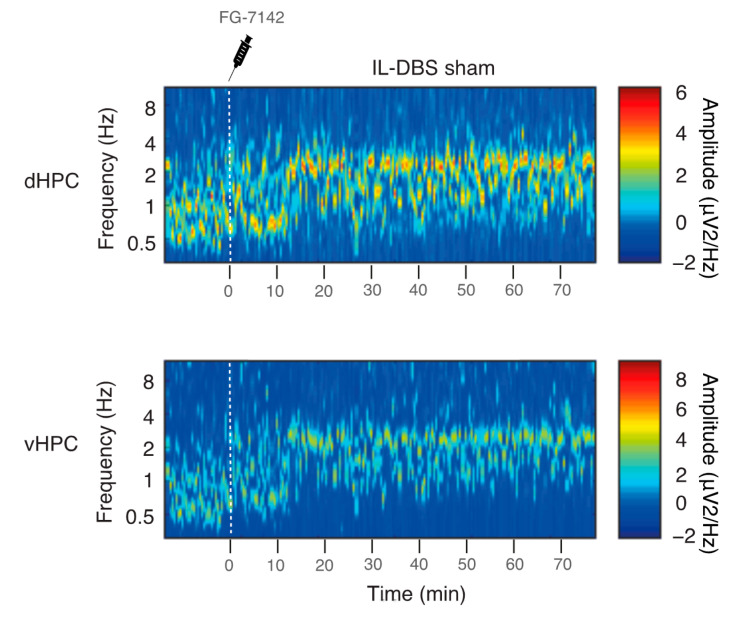
Profile of FG-7142 persistent effects in an IL-DBS sham-operated rat with electrodes inserted but in DBS-off mode during all of the experiments. Observe that spontaneous reversal was not apparent during the recorded time course.

One hour after surgery, recording began. The experimental procedure (Figure 1B) involved a continuous recording under anesthesia in the following conditions: (1) 300 s as baseline (henceforth, “basal” epoch); (2) 300 s following the administration of saline solution (“saline” epoch); (3) administration of the anxiogenic drug and, subsequently, recording for 15 min (“FG-7142” epoch); (4) 1 h of IL-DBS (bipolar stimulation at 130 Hz, 100 μA, and 80 μs) (“DBS” epoch); (5) 300 s in IL-DBS-off mode (“post-DBS” epoch). An additional group (6 animals with implanted electrodes) only received saline and FG-7142 but were in DBS-off mode during all the recording to visualize the evolution of the response to the drug and the intervention alone (Figure 2). 

LFPs were preamplified (model p511 AC Grass Preamplifier) and amplified (MPLI 4G21; Cibertec, Madrid, Spain), band-pass filtered (0.3–300 Hz), acquired, and digitized at 1 kHz (CED Micro; Cambridge Electronics Design, Cambridge, UK). Further details are reported elsewhere [67].

### 2.4. Histological Analysis 

After the experiments, animals were deeply anesthetized with an overdose of sodium pentobarbital (100 mg/kg, 20%; Dolethal, Vetoquinol, Madrid, Spain) and transcardially perfused with 500 mL of 0.1% heparinized saline (0.9%, pH 7), followed by 500 mL of 4% paraformaldehyde (Sigma-Aldrich, St. Louis, MO, USA). Brains were removed and maintained in 30% sucrose for cryopreservation. Coronal sections of 40 μm were obtained by a freezing microtome (Leica, Madrid, Spain), collected in the same solution, and stored for processing. Sections were then stained by the Giemsa technique to subsequently verify the placement of electrodes and the injection site (Figure 1C–G). Following verification, three animals with misplaced electrodes were excluded from the study.

### 2.5. Data Analysis 

All the analyses were performed offline with MATLAB software (The MathWorks, Natick, MA, USA; RRID:SCR_001622), using self-developed and built-in routines. First, LFPs were downsampled to 500 Hz, digitally notch-filtered with a Butterworth bandstop filter of around 50 Hz and harmonics until 200 Hz, and z-score normalized.

#### 2.5.1. Spectral Analysis

The recordings were analyzed based on the Fast Fourier Transform. Power spectral density was calculated by the Welch method, with a 5 s pwelch window with 50% overlap and nfft 1024, implemented with the Signal Processing MATLAB Toolbox. The analyzed bands were slow oscillations (SW < 1.5 Hz), delta (1.5–2.5 Hz), low theta (2.5–5 Hz), high theta (5–12 Hz), beta (16–30 Hz), low gamma (30–60 Hz), mid gamma (60–90 Hz), and high gamma (90–120 Hz). Spectral power and relative power were calculated for each band in consecutive 60 s windows to visualize the temporal evolution of each band. Relative power was calculated as band power/power in the 0–250 Hz.

#### 2.5.2. Wavelet Analysis

We used a continuous wavelet transform to improve the analysis on the time-frequency domain [102]. In addition, spectrograms and wavelet-based filters allowed us to better visualize the temporal evolution of the oscillations. 

Given that theta oscillations play a relevant role in hippocampal processing and within limbic structures, we analyzed this oscillation in further detail by detecting epochs with predominant low theta activity (“theta segments”). We defined theta segments as 2.5–5 Hz continuous oscillations constituting at least 30% of the total oscillatory activity for each time point and calculated in consecutive 60 s windows. The analysis of theta segments allowed the quantification of the temporal ratio (proportion of time with predominant theta segments/s), the mean duration, and the number of segments for each window. 

We also used the wavelet coefficient matrix to obtain coherograms for visualizing the coupling between channels. Phase-locking values (PLV) were defined as a measure of the stationarity of the phase differences in 10 s temporal windows and, therefore, of the phase-depending synchrony. The index used here was the weighted phase lag index (WPLI), in which the contribution of the observed phase leads and lags is weighted by the magnitude of the imaginary component of the cross-spectrum. This index increases the specificity by reducing the contribution of noise sources or volume conduction and the statistical power to detect changes in phase synchronization [103].

#### 2.5.3. Cross-Frequency Coupling

Amplitude–amplitude coupling was computed in RStudio (RRID:SCR_000432) as the Pearson linear correlation between the spectral power in two bands for the same channel. 

Phase–amplitude coupling (PAC) was measured in MATLAB through the modulation index (MI), as defined by Tort and colleagues [104] and described before [67]. The MI assumed normalized values between 0 and 1 and was considered statistically significant when its value was >2 SD of the substitute mean MI, constructed by 200 random permutations of the amplitude distribution.

### 2.6. Statistical Analysis 

The statistical analyses and data visualization were performed with RStudio. Recording periods (see Figure 1A) were grouped into the following: “basal” (first 300 s of the recording), “saline” (300 s before FG-7142 administration), “FG-7142” (300 s before the IL-DBS), five different periods during DBS treatment (DBS1, DBS2, DBS3, DBS4, DBS5), and “post-DBS” (300 s following the end of the IL-DBS). In each period, we analyzed a total time of 300 s composed of 5 consecutive 60 s windows. 

Given that the sample size was less than 30 and normal distribution could not be assumed, nonparametric tests were used. First, the variables were compared by Friedman’s chi-squared test with post hoc comparisons, with the Conover test for the pair-wise comparisons with the Bonferroni adjust method. A significance level of *p*-value < 0.05 was considered to determine the existence of statistically significant differences. For the Pearson linear correlation, significance levels were also obtained by the Ggally_cor and Ggpairs packages. 

## 3. Results

### 3.1. Spectral Analysis and Peak Frequency 

**Figure 3:**
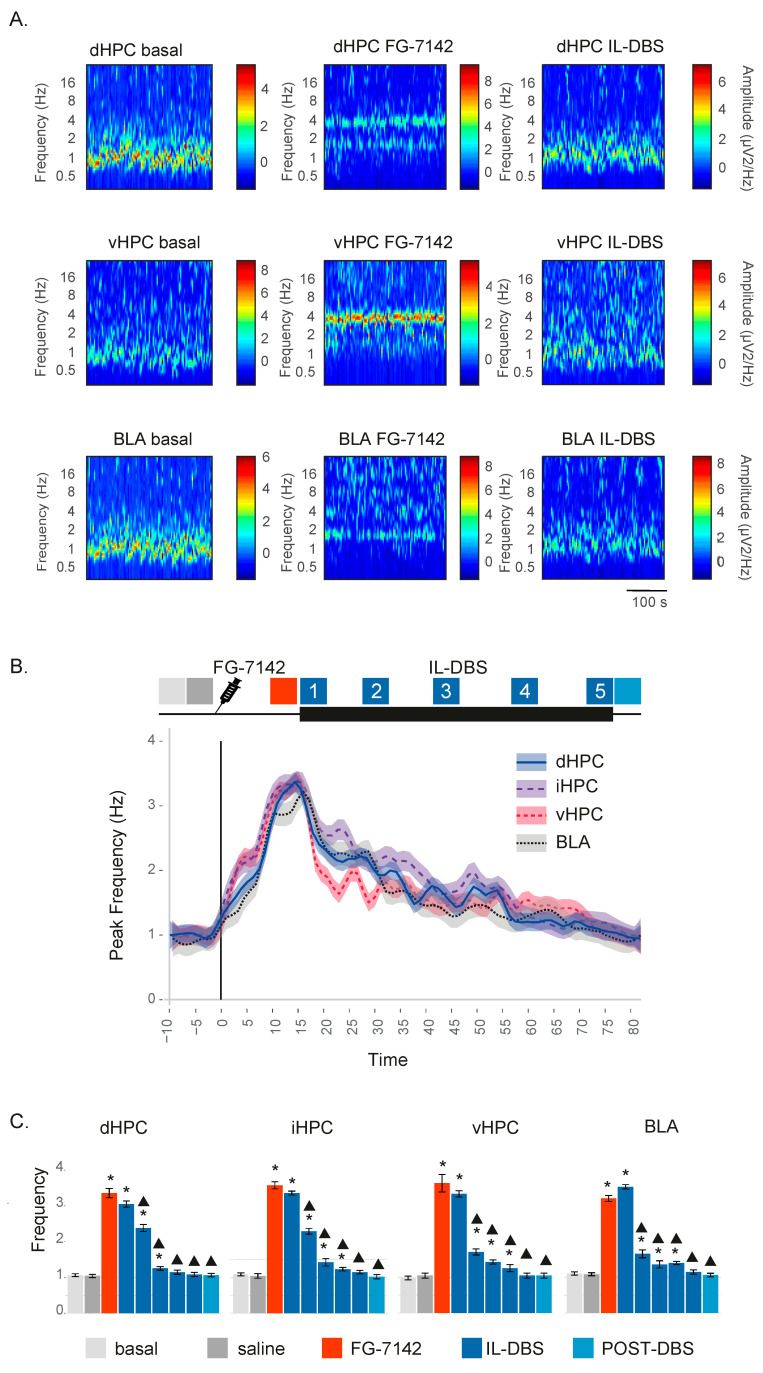
(**A**) Representative wavelet spectrograms in the dHPC, vHPC and BLA in basal conditions and after FG-7142 and IL_DBS interventions. Observe the increase in frequency induced by FG-7142 and the reversal of the oscillatory profile to slow waves by IL-DBS. (**B**) Time-course evolution of the peak frequency calculated by spectral decomposition. FG-7142 increases peak frequency to a similar value at 3–4 Hz in the low theta range, which is slightly lower in BLA. IL-DBS induces a progressive return to around 1 Hz, which is similar to basal values. (**C**) Statistical results of peak frequency in the 300 s periods indicated by color code in (**B**). * Statistical significance in pairwise comparisons with the basal period. Triangle: statistical significance in pairwise comparisons between IL-DBS and post-DBS with the FG-7142 period (degree of significance not indicated in order to better visualize results; please see Table 1). dHPC: dorsal hippocampus; iHPC: intermediate hippocampus; vHPC: ventral hippocampus; BLA: basolateral amygdala; IL: infralimbic cortex.

Urethane anesthesia was predominantly characterized by low-voltage oscillations below 2 Hz in the SW, and delta range, together with spontaneous short-lasting scattered faster waves. FG-7142 induced a long-lasting characteristic state, with a general switch to faster frequencies easily recognizable in all the recorded regions, and that lasted up to 2 h in sham-operate IL-DBS (Figure 2). The plateau period was typically observed between 10 and 15 min following administration. Details of a representative case with FG-7142 administration followed by IL-DBS are shown in Figure 3A. 

FG-7142 induced a marked and significant reduction in SW below 1.5 Hz in all regions, changing their oscillatory pattern to a regular oscillation with a robust peak frequency shifting to 3–3.5 Hz in the low theta range. IL-DBS progressively reversed the effect, reducing the peak frequency and returning to basal values approximately 45 min later. The effects of IL-DBS on peak frequency lasted in the post-stimulation period (Figure 3B,C). Statistical results are summarized in Table 1.

### 3.2. Relative Power of Slow Waves, Delta and Theta Band 

**Figure 4:**
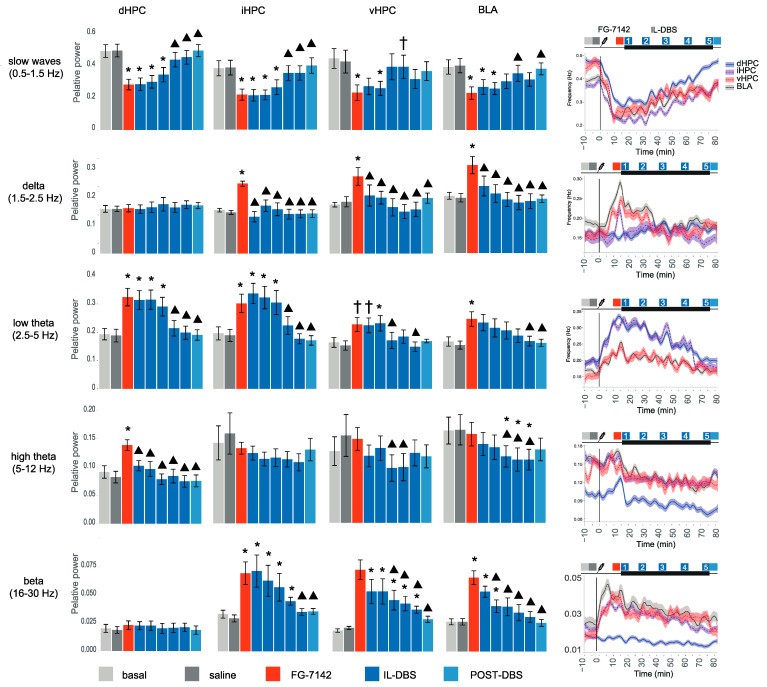
Statistical results of relative power of slow waves, delta, theta, and beta, calculated by spectral decomposition. * Statistical significance in pairwise comparisons with the basal period. Triangle: statistical significance in pairwise comparisons between IL-DBS and post-DBS with the FG-7142 period. Cross: statistical trend. Degree of significance not indicated to better visualize results; please see Supplementary Table S2. dHPC: dorsal hippocampus; iHPC: intermediate hippocampus; vHPC: ventral hippocampus; BLA: basolateral amygdala; IL: infralimbic cortex.

Figure 4 and Supplementary Table S2 summarize the statistical results of a Fourier analysis of the relative power for SW, delta, and theta bands. As mentioned previously, SW drastically diminished following the administration of FG-7142 in all regions. Interestingly, both sustained activity in the 1.5–2.5 Hz range (delta), and 16–30 Hz (beta) appeared in the BLA, vHPC, and iHPC, without any changes in the dHPC. The 2.5–5 Hz band (low theta) significantly increased in the dHPC, iHPC, and BLA, with a trend in vHPC. Again, 5–12 Hz (fast theta oscillations) only appeared in the dHPC after FG-7142. 

It is relevant to note that the increase observed in the relative power in most bands during the anxiety-like period was mainly due to the disappearance of the SW Delta oscillations, which also reduced their amplitude in this state; theta only increased its amplitude in the vHPC and iHPC, although its relative power was increased by the lack of SW. 

Essentially, in all cases, IL-DBS reversed the effect of FG-7142 administration over these oscillations in a time-dependent manner, returning to basal values during the stimulation and lasting in the post-stimulation period.

### 3.3. Theta Segments

**Figure 5:**
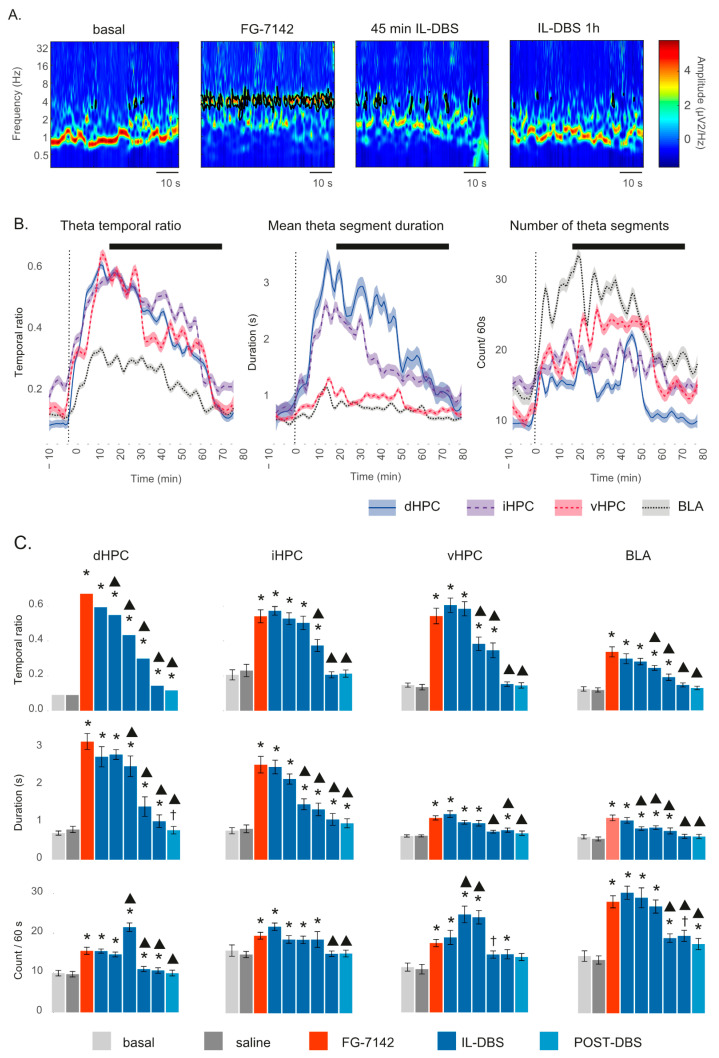
Theta segments: (**A**) vHPC: wavelet spectrograms. FG-7142 induces highly stable and long-lasting theta. IL-DBS disrupts theta segments, returning to basal values at 1 h. (**B**) Time course evolution of theta segments. Black line indicates the IL-DBS. (**C**) Statistical results of wavelet analysis. A more continuous theta is observed in the dHPC and iHPC, evidenced by a lower number of longer segments, while vHPC and BLA presented a more fractioned oscillation, indicated by more but shorter segments. IL-DBS first induced a disruption of the oscillations, represented by an increase in their number, together with shorter duration. Finally, theta segments diminished both in number and duration, eventually disappearing. Together, IL-DBS induced the progressive reduction in theta presence, as represented by a progressively lower temporal ratio. * Statistical significance in pairwise comparisons with the basal period. Triangle: statistical significance in pairwise comparisons with the FG-7142 period. Cross: statistical trend. Degree of significance not indicated to better visualize results; please see Supplementary Table S3. dHPC: dorsal hippocampus; iHPC: intermediate hippocampus; vHPC: ventral hippocampus; BLA: basolateral amygdala; IL: infralimbic cortex.

Theta activity is key for information processing within the hippocampus and other limbic structures [67], and it has even been proposed as a possible anxiety biomarker [105,106]. Thus, to better characterize the effects on the theta band, we further analyzed wavelet theta segments (Figure 5 and Supplementary Table S3). FG-7142 not only showed an increase in theta oscillation amplitudes but also their persistence over time (Figure 5A,B). The theta temporal ratio experienced a net increase in all hippocampal regions but to a lesser extent in BLA, where shorter segments were found (Figure 5B,C). IL-DBS decreased both the theta temporal ratio and mean duration (Figure 5B,C). As the IL-DBS advanced, a more fractionated theta could be appreciated (more segments of shorter duration), suggesting the discontinuation of theta activity. Finally, IL-DBS reduced both the number of segments and their duration, resulting in a progressive return to basal values of the temporal ratio. 

### 3.4. Effects of FG-7142 and IL-DBS on Local Gamma Power

FG-7142 administration induced an increase in mid gamma (60–90 Hz) power in the vHPC. High gamma (90–120 Hz) was also raised in the BLA and vHPC after treatment. Neither the iHPC nor the dHPC exhibited changes in gamma power in response to the anxiogenic drug. 

**Figure 6:**
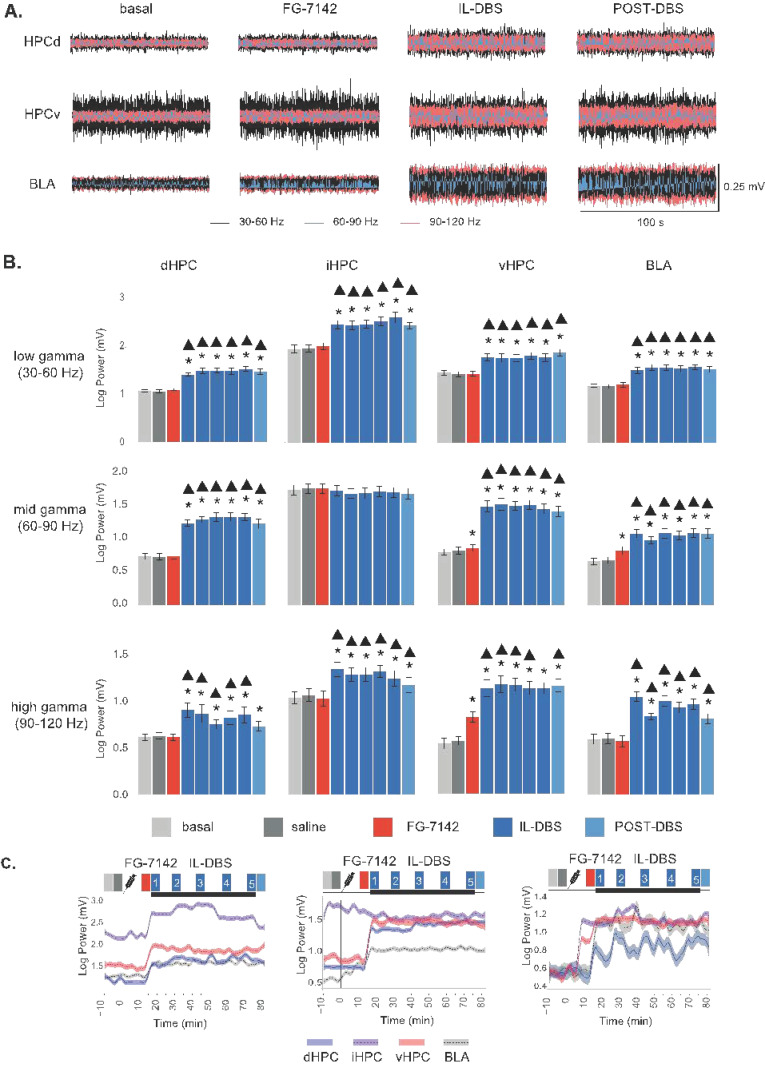
Effects of F-7142 and IL-DBS on gamma power: (**A**) wavelet-filtered oscillations showing the increase in all three gamma bands induced by IL-DBS. (**B**) Statistical results of gamma power computed by spectral decomposition. FG-7142 induces only statistical increases in vHPC and BLA, while a general increase in gamma power is observed during IL-DBS and post-DBS. * Statistical significance in pairwise comparisons with the basal period. Triangle: statistical significance in pairwise comparisons between IL-DBS and post-DBS with the FG-7142 period. Degree of significance not indicated to better visualize results; please see Supplementary Table S4. (**C**) Longitudinal evolution of gamma power. dHPC: dorsal hippocampus; iHPC: intermediate hippocampus; vHPC: ventral hippocampus; BLA: basolateral amygdala; IL: infralimbic cortex.

In contrast, the IL-DBS induced an increase in gamma power in the network, which also lasted during the post-DBS period. In brief, low and high gamma power increased in the vHPC, iHPC, dHPC, and BLA, compared to both the basal and the anxiety-like states. Mid gamma presented a similar pattern in the vHPC, dHPC, and BLA, but without any changes in the iHPC (Figure 6A,B and Supplementary Table S4).

### 3.5. Phase Synchronization

**Figure 7:**
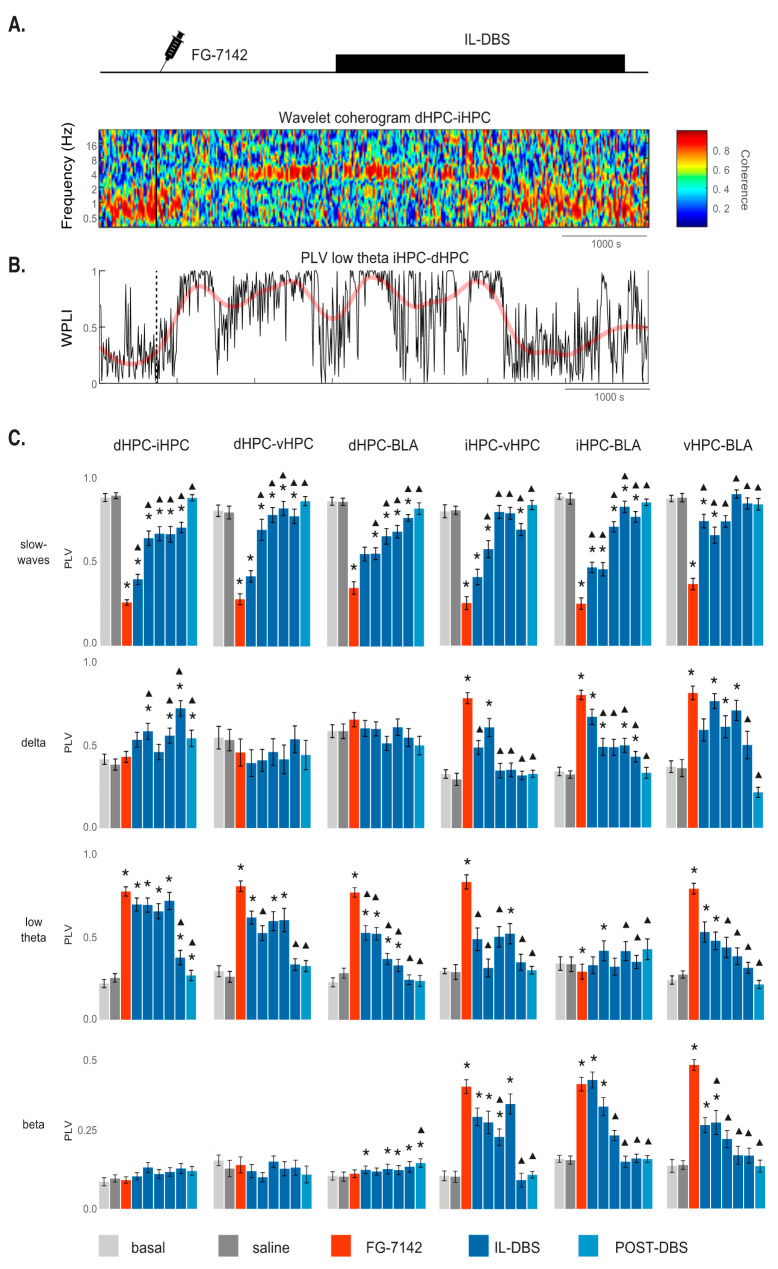
Effects on network synchronization induced by FG-7142 injection and IL-DBS: (**A**) A representative wavelet coherogram of dHPC-iHPC. At basal conditions, high coherence is observed at slow frequencies, which is typical of anesthesia. A clear increase in theta synchronization, together with a reduction in slow waves frequencies, can be observed in response to FG-7142. A return to basal values is induced by IL-DBS. (**B**) Time-course evolution of phase-locking value (PLV) measured as weighted phase lag index (WPLI) at low theta frequencies. WPLI values close to 1 indicate high phase synchronization at the selected frequency band (low theta in this case). Phase-locking under FG-7142 raises values close to 1. (**C**) Statistical results of WPLI. * Statistical significance in pairwise comparisons with the basal period. Triangle: statistical significance in pairwise comparisons with the FG-7142 period. Degree of significance not indicated to better visualize results; please see Supplementary Table S4. dHPC: dorsal hippocampus; iHPC: intermediate hippocampus; vHPC: ventral hippocampus; BLA: basolateral amygdala; IL: infralimbic cortex.

Phase-locking allowed us to assess the synchronization between structures at certain bands, measured as WPLI, to increase accuracy. Figure 7, Supplementary Figure S1, and Supplementary Table S5 show the results. In basal conditions (anesthesia), there was a high phase-locking in SW (mean PLV > 0.8; Figure 7A,C). However, FG-7142 increased the PLV at low theta frequencies between all the hippocampal subregions (dHPC–iHPC, iHPC–vHPC, dHPC–vHPC), as well as between the dHPC–BLA and vHPC–BLA (Figure 7B,C). The most ventral subregions (vHPC–BLA, iHPC–BLA, vHPC–iHPC) increased the PLV at both delta and beta frequencies. When the IL-DBS was applied (Figure 7B,C), the PLV at SW frequency progressively increased in all regions, reaching basal values during the stimulation or in the post-DBS period; this was sooner in the vHPC–BLA and vHPC–iHPC. The anxiety-evoked PLV at low theta frequencies returned to basal values with the IL-DBS, with shorter timings in the dHPC–BLA and iHPC–BLA pairs and longer timing in the vHPC–BLA. Both the anxiety-induced delta and beta PLV between the iHPC–vHPC, iHPC–BLA, and vHPC–BLA decreased with IL-DBS. In contrast, with the IL-DBS, delta increased in the dHPC–iHPC and beta increased in the dHPC–BLA. Therefore, anxiety increased communication at specific frequencies (theta, delta and beta) in different subnetworks, which IL-DBS reversed. 

### 3.6. Cross-Frequency Coupling 

Within each structure, changes in cross-frequency coupling were induced by FG-7142 and IL-DBS, both in amplitude–amplitude coupling and phase–amplitude coupling. 

#### 3.6.1. Amplitude–Amplitude Correlation 

**Figure 8:**
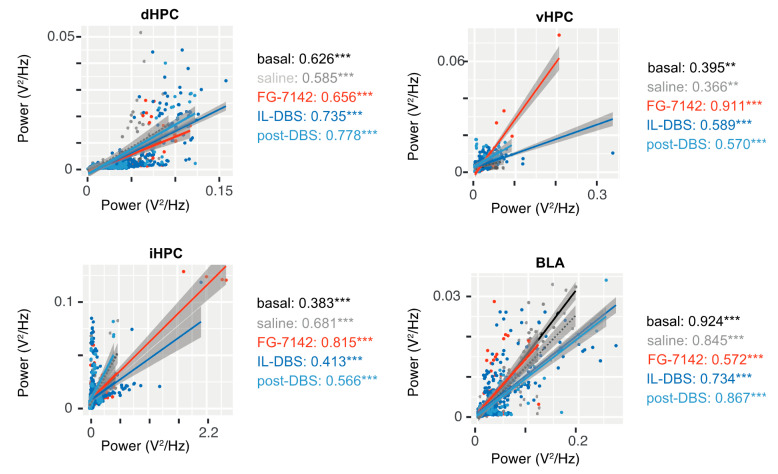
Amplitude–amplitude correlation between 2.5–5 Hz band and beta (16–30 Hz). Correlation at vHPC, and to a lesser extent iHPC, discriminates the effect of FG-7142 and IL-DBS well, with a high correlation following FG-7142 administration that was lower under the stimulation. r values are indicated in the right side; ** *p* < 0.01; *** *p* < 0.001. Other amplitude–amplitude correlations are represented in Supplementary Figure S2.

Both FG-7142 and IL-DBS produced changes in the amplitude–amplitude coupling between low-frequency oscillations in the 2.5–5 Hz range, beta activity (16–30 Hz), and gamma. Interestingly, a different pattern between regions was found (Figure 8). 

A topographic organization appeared, since the most evident results appeared in the vHPC and, to a lesser extent, the iHPC. In these regions, clearly different effects of the FG-7142 and DBS could be appreciated. FG-1742 generated a high correlation between the 2.5–5 Hz and faster oscillations. These effects were reversed by the IL-DBS (diminished slope), meaning similar 2.5–5 Hz amplitude values correlated with lower beta power. This pattern was less evident in the correlations calculated for dHPC and BLA areas. 

Remarkably, in response to FG-7142, the correlation between the 2.5–5 Hz band and beta activity (16–30 Hz) increased in the vHPC (r = 0.911; *p* < 0.001) and in the iHPC (r = 0.815; *p* < 0.001). After IL-DBS, and during post-DBS, this value decreased (vHPC r = 0.589, iHPC r = 0.413, *p* < 0.001; vHPC r = 0.570, iHPC r = 0.566, *p* < 0.001). In contrast, few changes were observed in the dHPC. Finally, the inverse pattern appeared in BLA, with the lowest correlation occurring during FG-7142 administration.

In the dHPC, the correlation between beta and low gamma diminished in the FG-7142, DBS, and post-DBS periods, while the correlation between low, mid, and high gamma increased. The correlation between mid and high gamma was always high (r > 0.99, *p* < 0.001). 

The iHPC and the vHPC exhibited the strongest correlations between the 2.5–5 Hz band and gamma during FG-7142 administration, though this diminished during IL-DBS. In this period, the iHPC showed the highest correlation between beta and mid gamma bands (r > 0.925, *p* < 0.001). The correlation between low and mid gamma bands was also high during the anxiogenic period in the vHPC (r > 0.917, *p* < 0.001). As in the dHPC in basal conditions, the correlation between mid and high gamma bands was high (r > 0.972, *p* < 0.001). However, in the vHPC, this correlation was reduced during IL-DBS (r > 0.686, *p* < 0.001). 

In the BLA, the strongest correlations between the 2.5–5 Hz and faster oscillations, as well as beta gamma correlations, appeared during the post-DBS period. Similar to the dHPC, the correlation between mid and high gamma bands was always extreme (r < 0.9). For more details, see Supplementary Figure S2. 

#### 3.6.2. Phase-Amplitude Coupling 

**Figure 9:**
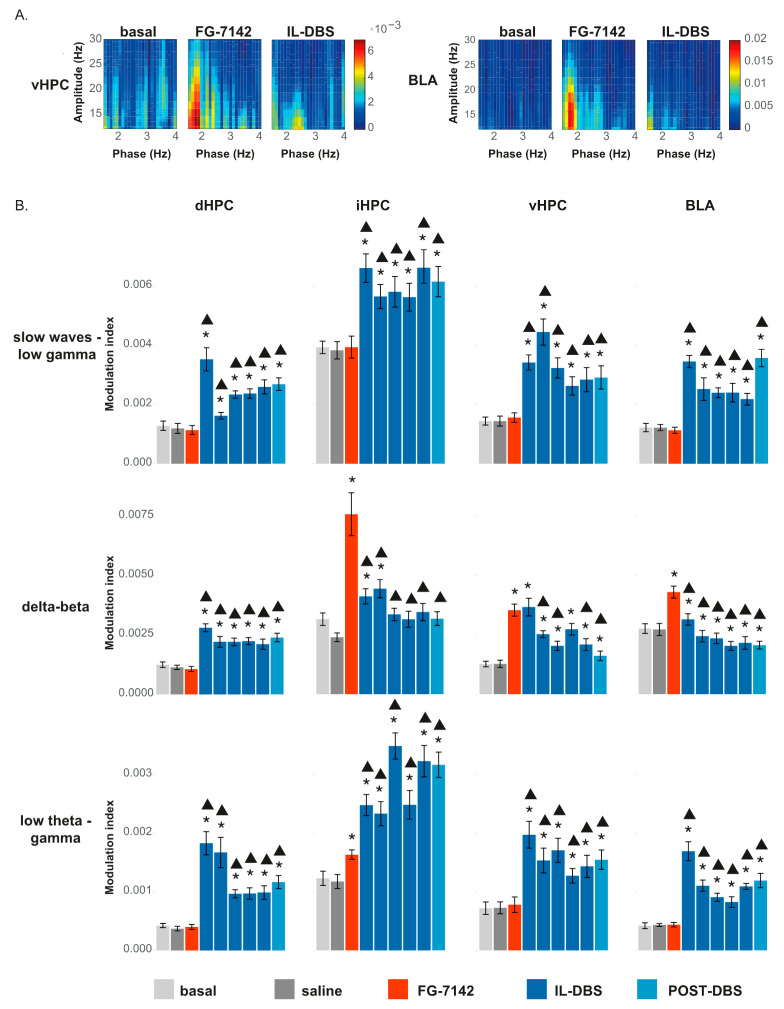
Effects of FG-7142 and IL-DBS over phase–amplitude coupling: (**A**) Comodulograms between 1–4 Hz and 10–30 Hz in vHPC and BLA showing an increase in coupling following administration of FG-7142 and a return to basal values with IL-DBS. (**B**) Statistical results of phase–amplitude coupling measured as the modulation index. * Statistical significance in pairwise comparisons with the basal period (degree of significance not indicated to better visualize results; please see Table S6). Triangle: statistical significance in pairwise comparisons between IL-DBS and post-DBS with the FG-7142 period. dHPC: dorsal hippocampus; iHPC: intermediate hippocampus; vHPC: ventral hippocampus; BLA: basolateral amygdala; IL: infralimbic cortex.

We obtained the MI between different bands in each structure to assess the PAC (Supplementary Table S6). Figure 9A displays a representative case in vHPC (Figure 9A, left) and BLA (Figure 9A, right). Statistical results, shown in Figure 9B, proved that FG-7142 increased delta–beta PAC in iHPC, vHPC, and BLA, as well as low theta–gamma coupling in the iHPC. However, delta–beta PAC remained similar to basal levels in the dHPC. 

IL-DBS reversed the changes observed in the delta–beta coupling, returning to basal values or even lower levels in BLA. In contrast, SW–low gamma and low theta–low gamma coupling were highest during the IL-DBS, as we have previously observed [67].

## 4. Discussion

Our study aimed to characterize oscillatory activity throughout the amygdala–hippocampal network under the effect of an anxiogenic drug, the inverse benzodiazepine FG-7142. Further, based on the high comorbidity between anxiety and depression, we analyzed the role of the IL-DBS, which exerts an antidepressant action in rats [39,40], as well as its equivalent in humans, the scACC DBS [15,27,107]. With this study, our purpose was to contribute to understandings around the neural circuits altered by anxiety, which is highly comorbid with other mental disorders, especially depression. Additionally, we aimed to shed light on how the DBS modulates these circuits. 

### 4.1. Study Limitations

We are aware that the main limitation of the present study resides in its use of urethane anesthesia, which makes it necessary to be cautious in the frequency range of the oscillations observed. However, studying neural oscillations under urethane anesthesia is easier than doing so under freely moving conditions, since the former allows stable recordings without behavioral-induced confounding effects (i.e., hippocampal theta activity is activated during locomotion, grooming, and many other behaviors). Additionally, urethane allows a more realistic approach to neural activity than other anesthetics, and has therefore been widely used for acute electrophysiological experiments to study brain oscillations and neuronal discharges. Under urethane anesthesia, spontaneous neural oscillations alternate in a cyclic sleep-like manner [108]. Nevertheless, since its anesthetic power is lower, it needs to be supplemented with local anesthesia. However, urethane typically induces large amplitude and slow frequency oscillations that are widely distributed in the neocortex and which are similar to the oscillatory patterns characteristic of non-REM sleep [109]. In the hippocampus, these oscillations induce an alternation between slow waves (in the slow oscillations and delta frequency ranges) and short spontaneous theta periods, as our group, as well as the work of others, has previously observed [67,110,111,112]. SW are widely present during anesthesia and thus cannot be assumed to be a basal physiological value. However, SW also appear in unanesthetized animals and are related to the antidepressant response induced by laughing gas (N_2_O), another NMDA receptor blocker [113]. Indeed, we point to this specific brain state, which is characterized by slow oscillations, as being responsive to the molecular changes related to fast-antidepressant responses. 

Additionally, our lack of behavioral tests could be suggested as a weakness. However, as described before, a large body of evidence supports the anxiogenic effects of FG-7142 in rodents, cats, primates, and even humans [84,90,114,115,116,117,118,119,120]. Similar doses of FG-7142 injected ip in rats induce anxious behaviors at timings that are compatible with the changes described in this article. 

In rodents, FG-7142 induces an increase in nonambulatory motor activity [78] but reduces exploratory behavior [80,81]. In the elevated plus-maze, FG-7142 reduces the exploration time spent in the open arms and increases the time spent in the closed arms [71]. Additionally, FG-7142 enhances behavioral suppression in response to safety signal withdrawal [82], exerts a proconflicting action, enhances shock-induced drinking suppression [79], and impairs fear extinction [83] and social interaction [84,85]. Its behavioral effects also include learned helplessness in a similar way to inescapable stress [84]. This fact is particularly relevant for our study about the IL-DBS mechanisms underlying its antidepressant actions, since learned helplessness constitutes a stress-based model of depression-like coping deficit in aversive but avoidable situations [121]. 

Regarding its neurobiological effects in rodents, FG-7142 also induces anxiety-related changes, including increases in corticosterone in plasma levels [86,87] and the activation of brain structures involved in stress, including the amygdala, the vHPC, the bed nucleus of the stria terminalis, and the cingulate, prelimbic, and IL cortices, as well as the brainstem nuclei of the reticular ascending arousal system, including the locus coeruleus, the periaqueductal grey, the nucleus incertus, and the dorsal raphe nucleus [78,79,96,97]. Moreover, Hackler and colleagues observed an increase in BOLD signal in the amygdala, dHPC, and hypothalamus for at least 40 min following FG-7142 administration (the authors did not report longer times) [85]. This effect is in accordance with what we observed in the sham-operated animals, without spontaneous reversal during the recording and 80 min after administration.

Additionally, the antidepressant- and anxiolytic-like action of IL-DBS in rats has been previously observed in freely behaving animals [39,40]. DBS applied at the ventromedial prefrontal cortex, and mainly restricted to the IL, reduces immobility time in the forced swimming test, which is used to evaluate learned helplessness, and increases climbing and swimming behaviors, which are usually considered as antidepressant-like actions. Additionally, DBS induces anxiolytic-like effects in the novelty-suppressed feeding test. 

Therefore, in this study, our purpose was not to validate previous results but to complement them in a pharmacological model of anxiety, with the description of the neural activity in the amygdala–hippocampal network using an anxiety-inducing validated tool [77]. Additionally, chose to minimize unnecessary animal suffering. 

However, further experiments in freely moving animals and studies in humans are necessary to confirm the usefulness of the studied parameters as anxiety biomarkers. 

### 4.2. Effects of FG-7142 and IL-DBS on the Spectral Composition

Specifically, in our study, FG-7142 administration drastically reduced SW power in all recorded areas. The treatment also increased the relative power of delta and beta oscillations in the iHPC, vHPC, and BLA, and low theta ratio in the dHPC, iHPC, and BLA, with a trend in the vHPC. Low theta power increased only in vHPC and iHPC. High theta relative power was only raised in the dHPC. Rises in mid or high gamma power were also found in both the BLA and the vHPC. 

Moreover, one hour of IL-DBS progressively reversed FG-7142 effects on SW, delta, theta, and beta, and was maintained in the DBS-off period. In contrast, gamma power was increased even more by the IL-DBS. In a previous study, we demonstrated that IL-DBS raises SW and gamma power in the dHPC and BLA [67] in anaesthetized animals. Additionally, in anaesthetized rats, Étievant and colleagues reported a similar effect in IL over slow oscillations after DBS, although they analyzed the full 0.25–4 Hz band [122]. We extend these previous results to a model of anxiety, showing the reversal of the pathological oscillatory profile. 

SW are travelling waves mainly generated in the mPFC [123,124]. Their high amplitude is related to widespread network synchronization [125,126,127] and might be critical to adapting to environmental pressures [128]. Furthermore, animal models [129,130] and clinical studies of depression [131,132,133] presented reduced SW activity. Accordingly, Duncan and colleagues proposed reduced sleep SW activity as biomarkers of depression [134] and increased in effective antidepressant therapies [135,136,137,138]. In this sense, our results supported that IL-DBS can be an efficient treatment to restore SW oscillations associated with anxiety.

On the other hand, theta oscillations are associated with active hippocampal states, allowing memory processing [139,140], with dissociable components for spatial and emotional cues [141]. Type 1 theta (fast theta) is related to locomotion and spatial processing, and is mainly detected in the dHPC [142,143]. In rodents, low theta or type 2 theta is expressed with immobility, arousal, and emotional behaviors [144], including stress [145,146,147,148], anxiety [148,149], and fear [150,151,152,153].

These different components of hippocampal theta are organized across the dorsoventral axis [154]. For example, in humans implanted with intracranially implanted electrodes, the posterior hippocampus (equivalent to dHPC) displays high theta oscillations (~8 Hz) related to spatial processing. In comparison, the anterior hippocampus (equivalent to vHPC) exhibits low theta activity (~3 Hz) during non-spatial cognitive processing [155]. Additionally, optogenetic studies in rats have shown that the vHPC drives type 2 theta [156]. 

The reduction in hippocampal activity is related to anxiolytic responses. An early but extensive review by Gray and McNaughton referred to an improvement in active avoidance in conflict-generating tasks [157]. In general, hippocampal lesions result in reduced anxiety and behavioral disinhibition. More precisely, the vHPC is involved in the control of behavior under anxiogenic situations [53]. A decrease in vHPC and BLA theta has been known to occur with familiarization to a novel environment in control rats, but not in stressed rats [147], while theta power increases in the vHPC, BLA and to a lesser extent in the dHPC in stressed rats compared to control rats during the exploration of open arms in the elevated plus-maze [148]. Thus, the lowering of theta in these areas is related to a decreased anxiety level. Our results are in line with this fundamental role.

In specific behaviors involving decisions related to conflict, hippocampal theta oscillations have been proposed as anxiety biomarkers by McNaughton and colleagues [106,158,159]. In addition, drugs with anxiolytic effects reduce reticular elicited hippocampal rhythms [158,160]. 

Accordingly, in our study, FG-7142 induced a sustained low theta, whereas the IL-DBS first discontinued this oscillation and finally suppressed it. We hypothesize that, under anxiety, the amygdala hijacks the hippocampus in a continuous search for stress resolution, which would be reflected as a sustained theta and could compromise standard cognitive processing. IL-DBS can shortcut this sustained and pathological theta, as supported by our wavelet analyses, leaving the hippocampus available for cognitive processing. 

We also found a reliable increase in gamma activity under IL-DBS, with only a moderate increase in mid and high gamma during FG-7142 administration. Fast-frequency oscillations reflect local neuronal assemblies [161,162,163]. Alterations in gamma have been described in mood disorders [164,165,166] and proposed as putative biomarkers of depression [167]. Our study agrees with animal and clinical studies that have reported similar increases in gamma power following acute ketamine administration at therapeutic levels [168,169,170,171,172,173]. Similarly to DBS, subanesthetic doses of ketamine, a noncompetitive *N*-methyl-d-aspartate (NMDA) receptor antagonist, also provides a fast antidepressant action [174], being particularly effective in anxious depression [175] and it is also helpful in relieving social anxiety disorder [176]. Cornwell and colleagues found that this rise in gamma power following ketamine administration, measured in cortical magnetoencephalographic recordings, is correlated to antidepressant responses and increased in responders vs. non-responders [177]. The increase in gamma power is more remarkable in depressive patients than in healthy controls. Even those exhibiting lower baseline gamma had better antidepressant action correlated to a higher increase in gamma power [178]. Gilbert and colleagues recently interpreted this influence in cortical networks as a therapeutic effect of modulating activation/inhibition levels while maintaining them at homeostatic levels [168]. However, most of these studies focused on cortical gamma and not hippocampal or amygdala activity. Other than gamma power, ketamine also induces antidepressant- and anxiolytic-like effects and reduces the reticular-elicited hippocampal theta in animal models [179]. Thus, IL-DBS and ketamine seem to share some electrophysiological features as fast antidepressant therapies. 

### 4.3. Communication in the Amygdala–Hippocampal Network

Coherent oscillatory activity in distributed networks is involved in information processing [180,181]. Our study also found a decreased synchronization of SW in all regions under the anxiety-like period. We have also shown an increase in the PLV at delta and beta frequencies between the iHPC–vHPC, iHPC–BLA, and vHPC–BLA. There was a low theta band within all the hippocampal areas studied, and between dHPC–BLA, and it increased in vHPC–BLA but decreased in iHPC–BLA. 

Taken together, the results suggest that, under the effects of FG-7142, the ventral subnetwork communicates via delta, low theta, and beta frequencies, while the intra-hippocampal communication affecting the dHPC operates only in the low theta range. 

An increased WPLI at theta frequencies in the resting state of individuals suffering generalized social anxiety disorder has recently been described [182]. Additionally, according to an hyperanxiety animal model of chronic unpredictable stress, theta coherence between vHPC–BLA was higher when avoiding open arms than in approach actions in the elevated plus-maze [148]. 

The enhancement observed in 2.5–5 Hz and beta amplitude–amplitude correlation, specifically in the vHPC, complements these results, supporting the proposal of the delta–beta correlation as a putative anxiety biomarker [183,184,185] and extending the possibility of evaluating the PAC within these frequencies. However, it is important to be cautious in these findings’ translation to human studies, since the frequency ranges differ slightly from those measured in anaesthetized rodents, in which slow rhythmical activity correspondent to theta rhythm occurs at lower frequencies [186]. Therefore, we have deliberately avoided delta or theta terms in the correlation results to reduce misinterpretations. 

Finally, in the analysis of phase–amplitude coupling, we found an increase between SW–gamma, delta–beta, and low theta–gamma coupling following IL-DBS. We already described this increase in cross-frequency coupling between SW and faster frequencies in the dHPC and BLA in anaesthetized rats following IL-DBS, without any anxiety model [67]. 

PAC reflects the coordination of local (fast) and widespread (slow) oscillations, allowing better integration of information in a neuronal network [187]. Indeed, Salimpour and colleagues recommended PAC-oriented neuromodulation as being useful in neurological disorders [188]. In the hippocampus, theta–gamma coupling has been widely involved in memory processing [189,190,191]. In this case, hippocampal and BLA local activity could be better integrated following IL-DBS, contributing to the therapeutic effect. 

## 5. Conclusions

In conclusion, our study characterizes the oscillations that emerge as a fingerprint in response to the anxiogenic drug FG-7142 in urethane-anesthetized rats. Additionally, our results provide evidence that IL-DBS reverses abnormal oscillatory processing in communication in the amygdala–hippocampal network. Further studies in freely moving animals and humans are encouraged in order to describe the oscillatory biomarkers of anxiety and to further analyze the effects of IL-DBS. 

## Figures and Tables

**Table 1:** Peak frequency computed by spectral decomposition.

Period	dHPC	iHPC	vHPC	BLA
**Basal**	1.063 ± 0.039	1.084 ± 0.042	0.979 ± 0.051	1.105 ± 0.045
**Saline**	1.042 ± 0.046	1.041 ± 0.063	1.048 ± 0.069	1.084 ± 0.042
**FG-7142**	3.355 ± 0.128 ***	3.574 ± 0.100 ***	3.629 ± 0.244 ***	3.202 ± 0.083 ***
**DBS1**	3.047 ± 0.087 ***	3.356 ± 0.056 ***	3.330 ± 0.087 ***	3.526 ± 0.061 ***
**DBS2**	2.380 ± 0.098 ***	2.292 ± 0.081 ***	1.705 ± 0.085 ***	1.653 ± 0.109 ***
**DBS3**	1.251 ± 0.051 ***	1.421 ± 0.101 ***	1.426 ± 0.061 ***	1.359 ± 0.101 **
**DBS4**	1.147 ± 0.058	1.230 ± 0.051	1.253 ± 0.098 **	1.398 ± 0.046 ***
**DBS5**	1.083 ± 0.060	1.147 ± 0.049	1.048 ± 0.070	1.147 ± 0.058
**POST-DBS**	1.063 ± 0.049	1.020 ± 0.060	1.048 ± 0.069	1.063 ± 0.049

Note: mean ± se (bold: statistical significance in pairwise comparisons to basal period; *** *p* < 0.001, ** *p* < 0.01).

## Data Availability

Data available on request.

## Supplementary Materials

The following are available online at https://www.mdpi.com/article/10.3390/biomedicines9070783/s1, Figure S1: Longitudinal analysis of Phase-locking value (WPLI), Figure S2: Cross-frequency amplitude-amplitude coupling, Table S1: Penetration coordinates in the recording areas, Table S2: Relative power, Table S3: Theta segments, Table S4: Gamma power, Table S5: Phase-locking value (WPLI), Table S6: Phase-amplitude coupling.
