# Supplementary material for: The Oscillatory Profile Induced by the Anxiogenic Drug FG-7142 in the Amygdala–Hippocampal Network Is Reversed by Infralimbic Deep Brain Stimulation: Relevance for Mood Disorders
Source: Biomedicines. 2021 Jul 6;9(7):783. doi: 10.3390/biomedicines9070783 (PMC8301458; doi:10.3390/biomedicines9070783)
Supplement: Supplementary file 1 [file biomedicines-09-00783-s001.zip › Biomedicines supplemental/SupplTable S1.pdf]

**Table S1**. Penetration coordinates in the recording areas.

| Region | Anterioposterior | Lateral | Dorsoventral |
|---|---|---|---|
| **dHPC** | -3.4 mm | 2.5 mm | 2.4 mm |
| **iHPC** | -5.8 mm | 5.8 mm | 5 mm |
| **vHPC** | -4.7 mm | 5 mm | 8.7 mm |
| **BLA** | -2.3 mm | 5 mm | 8.5 mm |

| Rat # | Region | Penetration angle | Rat # | Region | Penetration angle | Rat # | Region | Penetration angle |
|---|---|---|---|---|---|---|---|---|
| **1** | **dHPC** | 0º | **9** | **dHPC** | 0º | **17** | **dHPC** | 0º |
| | **iHPC** | 0º | | **iHPC** | 0º | | **iHPC** | 0º |
| | **vHPC** | 10º from midline | | **vHPC** | 0º | | **vHPC** | 0º |
| | **BLA** | 0º | | **BLA** | 10º from midline | | **BLA** | 10º from front |
| **2** | **dHPC** | 0º | **10** | **dHPC** | 0º | **18** | **dHPC** | 0º |
| | **iHPC** | 10º from midline | | **iHPC** | 0º | | **iHPC** | 10º from midline |
| | **vHPC** | 0º | | **vHPC** | 10º from midline | | **vHPC** | 0º |
| | **BLA** | 0º | | **BLA** | 0º | | **BLA** | 0º |
| **3** | **dHPC** | 0º | **11** | **dHPC** | 0º | **19** | **dHPC** | 30º from lateral |
| | **iHPC** | 0º | | **iHPC** | 10º from midline | | **iHPC** | 0º |
| | **vHPC** | 0º | | **vHPC** | 0º | | **vHPC** | 0º |
| | **BLA** | 10º from midline | | **BLA** | 0º | | **BLA** | 0º |
| **4** | **dHPC** | 10º from midline | **12** | **dHPC** | 10º from midline | **20** | **dHPC** | 0º |
| | **iHPC** | 0º | | **iHPC** | 0º | | **iHPC** | 10 from midline |
| | **vHPC** | 0º | | **vHPC** | 0º | | **vHPC** | 0º |
| | **BLA** | 0º | | **BLA** | 0º | | **BLA** | 0º |
| **5** | **dHPC** | 0º | 13 | **dHPC** | 0º | **21** | **dHPC** | 0º |
| | **iHPC** | 0º | | **iHPC** | 0º | | **iHPC** | 0º |
| | **vHPC** | 0º | | **vHPC** | 0º | | **vHPC** | 0º |
| | **BLA** | 20º from midline | | **BLA** | 0º | | **BLA** | 10º from front |
| **6** | **dHPC** | 0º | **14** | **dHPC** | 10º lateral | **22** | **dHPC** | 10º from midline |
| | **iHPC** | 0º | | **iHPC** | 0º | | **iHPC** | 0º |
| | **vHPC** | 20º from midline | | **vHPC** | 0º | | **vHPC** | 0º |
| | **BLA** | 0º | | **BLA** | 0º | | **BLA** | 0º |
| **7** | **dHPC** | 0º | **15** | **dHPC** | 0º | **23** | **dHPC** | 0º |
| | **iHPC** | 20º from midline | | **iHPC** | 10º from behind | | **iHPC** | 0º |
| | **vHPC** | 0º | | **vHPC** | 0º | | **vHPC** | 10º from midline |
| | **BLA** | 0º | | **BLA** | 0º | | **BLA** | 0º |
| **8** | **dHPC** | 20º from midline | **16** | **dHPC** | 0º | **24** | **dHPC** | 0º |
| | **iHPC** | 0º | | **iHPC** | 0º | | **iHPC** | 0º |
| | **vHPC** | 0º | | **vHPC** | 10º from midline | | **vHPC** | 0º |
| | **BLA** | 0º | | **BLA** | 0º | | **BLA** | 10º from midline |
