# Supplementary material for: The Oscillatory Profile Induced by the Anxiogenic Drug FG-7142 in the Amygdala–Hippocampal Network Is Reversed by Infralimbic Deep Brain Stimulation: Relevance for Mood Disorders
Source: Biomedicines. 2021 Jul 6;9(7):783. doi: 10.3390/biomedicines9070783 (PMC8301458; doi:10.3390/biomedicines9070783)
Supplement: Supplementary file 1 [file biomedicines-09-00783-s001.zip › Biomedicines supplemental/SupplTable S2.pdf]

Table S2. Relative power computed by spectral decomposition.

Band	Region	Basal	Saline	FG-7142	DBS1	DBS2	DBS3	DBS4	DBS5	POST-DBS
**Slow Waves**	**dHPC**	0.489 ± 0.040	0.492 ± 0.039	**0.281 ± 0.033*****	**0.284 ± 0.038*****	**0.298 ± 0.039*****	**0.342 ± 0.045*****	0.436 ± 0.045	0.450 ± 0.043	0.491 ± 0.038
	**iHPC**	0.380 ± 0.047	0.384 ± 0.046	**0.217 ± 0.034*****	**0.212 ± 0.036*****	**0.215 ± 0.031*****	**0.262 ± 0.046*****	0.351 ± 0.046	0.350 ± 0.045	0.397 ± 0.048
	**vHPC**	0.443 ± 0.061	0.424 ± 0.071	**0.232 ± 0.048****	0.270 ± 0.051	**0.258 ± 0.044****	0.392 ± 0.081	0.392 ± 0.072	0.315 ± 0.059	0.365 ± 0.058
	**BLA**	0.390 ± 0.044	0.398 ± 0.044	**0.229 ± 0.038*****	**0.266 ± 0.046***	**0.256 ± 0.037***	0.301 ± 0.045	0.351 ± 0.050	0.313 ± 0.042	0.379 ± 0.037
**Delta**	**dHPC**	0.158 ± 0.012	0.158 ± 0.010	0.160 ± 0.013	0.157 ± 0.015	0.164 ± 0.019	0.174 ± 0.024	0.163 ± 0.018	0.173 ± 0.014	0.170 ± 0.011
	**iHPC**	0.154 ± 0.006	0.146 ± 0.007	**0.248 ± 0.009*****	0.130 ± 0.018	0.169 ± 0.020	0.157 ± 0.023	0.140 ± 0.017	0.140 ± 0.014	0.141 ± 0.014
	**vHPC**	0.173 ± 0.008	0.183 ± 0.018	**0.273 ± 0.031*****	0.205 ± 0.037	0.198 ± 0.023	0.164 ± 0.031	0.148 ± 0.025	0.156 ± 0.026	0.196 ± 0.018
	**BLA**	0.202 ± 0.012	0.195 ± 0.015	**0.311 ± 0.031****	0.237 ± 0.035	0.210 ± 0.031	**0.290 ± 0.025****	**0.179 ± 0.025*****	**0.183 ± 0.026****	0.192 ± 0.013
**Low Theta**	**dHPC**	0.193 ± 0.020	0.188 ± 0.023	**0.324 ± 0.031*****	**0.314 ± 0.033*****	**0.315 ± 0.034*****	**0.290 ± 0.033****	0.214 ± 0.028	0.198 ± 0.023	0.190 ± 0.019
	**iHPC**	0.196 ± 0.023	0.189 ± 0.021	**0.302 ± 0.033*****	**0.337 ± 0.036*****	**0.323 ± 0.040*****	**0.305 ± 0.043****	0.223 ± 0.032	0.177 ± 0.018	0.171 ± 0.017
	**vHPC**	0.164 ± 0.017	0.153 ± 0.016	**0.227 ± 0.025†**	**0.224 ± 0.026***	**0.231 ± 0.027****	0.171 ± 0.029	0.184 ± 0.023	0.149 ± 0.017	0.169 ± 0.006
	**BLA**	0.167 ± 0.016	0.154 ± 0.014	**0.246 ± 0.026***	0.233 ± 0.029	0.215 ± 0.032	0.206 ± 0.030	0.187 ± 0.024	0.168 ± 0.017	0.162 ± 0.013
**High Theta**	**dHPC**	0.091 ± 0.011	0.082 ± 0.010	**0.139 ± 0.010*****	0.102 ± 0.009	0.097 ± 0.013	0.079 ± 0.009	0.084 ± 0.012	0.075 ± 0.010	0.076 ± 0.011
	**iHPC**	0.142 ± 0.030	0.159 ± 0.037	**0.133 ± 0.010****	0.124 ± 0.012	0.114 ± 0.012	0.116 ± 0.016	0.113 ± 0.014	0.108 ± 0.015	0.130 ± 0.020
	**vHPC**	0.127 ± 0.025	0.155 ± 0.037	0.148 ± 0.020	0.118 ± 0.019	0.132 ± 0.022	0.097 ± 0.023	0.098 ± 0.024	0.124 ± 0.023	0.118 ± 0.020
	**BLA**	0.163 ± 0.024	0.165 ± 0.027	0.157 ± 0.021	0.140 ± 0.020	0.134 ± 0.022	**0.118 ± 0.019***	**0.112 ± 0.021***	**0.112 ± 0.018***	0.130 ± 0.020
**Beta**	**HPCD**	0.020 ± 0.004	0.019 ± 0.0003	0.023 ± 0.004	0.022 ± 0.004	0.022 ± 0.004	0.020 ± 0.004	0.020 ± 0.004	0.021 ± 0.004	0.019 ± 0.004
	**HPCI**	0.033 ± 0.004	0.029 ± 0.0003	**0.069 ± 0.010*****	**0.071 ± 0.014*****	**0.062 ± 0.014*****	**0.057 ± 0.012*****	**0.044 ± 0.003*****	0.035 ± 0.003	0.035 ± 0.003
	**HPCV**	0.018 ± 0.002	0.021 ± 0.0001	**0.072 ± 0.009*****	**0.053 ± 0.011*****	**0.053 ± 0.011*****	**0.045 ± 0.009*****	**0.042 ± 0.006*****	**0.037 ± 0.003*****	0.028 ± 0.003
	**BLA**	0.026 ± 0.003	0.026 ± 0.0003	**0.065 ± 0.006*****	**0.053 ± 0.005*****	**0.040 ± 0.006***	0.040 ± 0.008	0.034 ± 0.007	0.030 ± 0.005	0.035 ± 0.003

Note: Mean ± se (bold: in pairwise comparisons to basal period; ***p< 0.001, **p< 0.01, *p< 0.05, †: 0.08<p<0.05)
