# Supplementary material for: The Oscillatory Profile Induced by the Anxiogenic Drug FG-7142 in the Amygdala–Hippocampal Network Is Reversed by Infralimbic Deep Brain Stimulation: Relevance for Mood Disorders
Source: Biomedicines. 2021 Jul 6;9(7):783. doi: 10.3390/biomedicines9070783 (PMC8301458; doi:10.3390/biomedicines9070783)
Supplement: Supplementary file 1 [file biomedicines-09-00783-s001.zip › Biomedicines supplemental/SupplTable S3.pdf]

Table S3. Analysis of theta segments parameters by wavelet analysis

Parameter	Region	Basal	Saline	FG-7142	DBS1	DBS2	DBS3	DBS4	DBS5	POST-DBS
Temporal Ratio	**dHPC**	0.0913 ± 0.0070	0.0904 ± 0.0073	**0.6709± 0.0507*** **	**0.5963 ± 0.0501*** **	**0.5491 ± 0.0511*** **	**0.4339 ± 0.0304*** **	**0.2987 ± 0.0356*** **	**0.1439 ± 0.0126*** **	**0.1170 ± 0.0127* **
	**iHPC**	0.2061 ± 0.0289	0.2309 ± 0.0352	**0.5417 ± 0.0370*** **	**0.5730 ± 0.0247*** **	**0.5283± 0.0335*** **	**0.5039 ± 0.0383*** **	**0.3743 ± 0.0340*** **	0.2061 ± 0.0175	0.2135 ± 0.0206
	**vHPC**	0.1473 ± 0.0119	0.1353 ± 0.0158	**0.5433 ± 0.0447*** **	**0.6053 ± 0.0412*** **	**0.5847 ± 0.0409*** **	**0.3840 ± 0.0382*** **	**0.3467 ± 0.0422*** **	0.1527 ± 0.0130	0.1423 ± 0.0153
	**BLA**	0.1230 ± 0.0127	0.1174 ± 0.0120	**0.3357 ± 0.0289*** **	**0.2961 ± 0.0286*** **	**0.2800 ± 0.0186*** **	**0.2435 ± 0.0137*** **	**0.1904 ± 0.0182*** **	0.1461 ± 0.0115	0.1287 ± 0.0100
Mean Width	**dHPC**	0.7109 ± 0.0552	0.8070 ± 0.0837	**3.1604 ± 0.2137*** **	**2.7522 ± 0.2710*** **	**2.8117 ± 0.1298*** **	**2.4991 ± 0.2755*** **	**1.4204 ± 0.2609*** **	**1.0287 ± 0.1696** **	0.7887 ± 0.0996
	**iHPC**	0.7745 ± 0.0824	0.8248 ± 0.1043	**2.5422 ± 0.2227*** **	**2.4791 ± 0.1841*** **	**2.1570 ± 0.1357*** **	**1.4822 ± 0.1438*** **	**1.3427 ± 0.1690*** **	**1.0764 ± 0.1580* **	**0.9736 ± 0.1217* **
	**vHPC**	0.6340 ± 0.0297	0.6373 ± 0.0274	**1.1180 ± 0.0564*** **	**1.2180 ± 0.0887*** **	**1.0013 ± 0.0502*** **	**0.9747 ± 0.0762*** **	0.7480 ± 0.0373	**0.7900 ± 0.0556* **	0.7060 ± 0.0605
	**BLA**	0.6096 ± 0.0544	0.5552 ± 0.0528	**1.1183 ± 0.0762*** **	**1.1059 ± 0.0770*** **	**0.8362 ± 0.0473*** **	**0.8565 ± 0.0506*** **	**0.7670 ± 0.0808*** **	0.6257 ± 0.0538	0.6200 ± 0.0567
Segments	**dHPC**	9.897 ± 0.696	9.636 ± 0.696	**15.530 ± 0.892*** **	**15.470 ± 0.527*** **	**14.617 ± 0.608*** **	**21.487 ± 1.067*** **	**10.939 ± 0.643** **	**10.565 ± 0.686* **	9.929 ± 0.736
	**iHPC**	15.826 ± 1.428	14.826 ± 0.790	**19.574 ± 0.866*** **	**21.835 ± 0.938*** **	**18.614 ± 0.961** **	**18.522 ± 0.954* **	**18.609 ± 1.986* **	15.017 ± 0.699	**15.087 ± 0.857* **
	**vHPC**	11.427 ± 1.019	10.907 ± 1.217	**17.483 ± 0.0911*** **	**18.920± 1.771*** **	**24.707 ± 2.103*** **	**24.000 ± 1.696*** **	**14.600 ± 0.907†**	**14.667 ± 1.1183* **	13.960 ± 0.890
	**BLA**	14.435 ± 1.323	13.443 ± 1.036	**28.130 ± 0.1.529*** **	**30.417 ± 1.657*** **	**29.139 ± 2.500*** **	**26.930 ± 1.645*** **	**18.974 ± 1.071†**	**19.530 ± 1.406*** **	17.470 ± 1.409

Note: Mean ± se (bold: statistical significance in pairwise comparisons to basal period; ***p< 0.001, **p< 0.01; *p< 0.05; †: 0.08<p<0.05)
