# Supplementary material for: The Oscillatory Profile Induced by the Anxiogenic Drug FG-7142 in the Amygdala–Hippocampal Network Is Reversed by Infralimbic Deep Brain Stimulation: Relevance for Mood Disorders
Source: Biomedicines. 2021 Jul 6;9(7):783. doi: 10.3390/biomedicines9070783 (PMC8301458; doi:10.3390/biomedicines9070783)
Supplement: Supplementary file 1 [file biomedicines-09-00783-s001.zip › Biomedicines supplemental/SupplTable S4.pdf]

Table S4. Gamma power.

Band		Basal	Saline	FG-7142	DBS1	DBS2	DBS3	DBS4	DBS5	POST-DBS
**Low Gamma**	**dHPC**	1.205 ± 0.024	1.196 ± 0.033	1.236 ± 0.026	**1.590 ± 0.040*** **	**1.679 ± 0.066*** **	**1.684 ± 0.059*** **	**1.723 ± 0.064*** **	**1.651 ± 0.065*** **	**1.521 ± 0.056*** **
	**iHPC**	2.208 ± 0.084	2.258 ± 0.080	2.570 ± 0.097	**2.752 ± 0.100*** **	**2.780 ± 0.0.98*** **	**2.848 ± 0.101*** **	**2.939 ± 0.129*** **	**2.747 ± 0.078*** **	**2.596 ± 0.078*** **
	**vHPC**	1.641 ± 0.060	1.609 ± 0.061	1.620 ± 0.055	**2.007 ± 0.081*** **	**1.991 ± 0.095*** **	**1.989 ± 0.084*** **	**2.040 ± 0.081*** **	**2.000 ± 0.093*** **	**2.114 ± 0.081*** **
	**BLA**	1.332 ± 0.041	1.319 ± 0.047	1.357 ± 0.056	**1.700 ± 0.074*** **	**1.761 ± 0.073*** **	**1.753 ± 0.065*** **	**1.732 ± 0.068*** **	**1.771 ± 0.059*** **	**1.719 ± 0.071*** **
**Mid Gamma**	**dHPC**	0.744 ± 0.047	0.737 ± 0.047	0.741 ± 0.041	**1.247 ± 0.052*** **	**1.298 ± 0.045*** **	**1.340 ± 0.064*** **	**1.341 ± 0.064*** **	**1.338 ± 0.060*** **	**1.240 ± 0.072*** **
	**iHPC**	1.747 ± 0.075	1.768 ± 0.072	1.772 ± 0.073	1.737 ± 0.076	1.695 ± 0.073	1.698 ± 0.081	1.724 ± 0.080	1.712 ± 0.075	1.694 ± 0.080
	**vHPC**	0.801 ± 0.046	0.822 ± 0.052	**0.861 ± 0.050*** **	**1.494 ± 0.087*** **	**1.525 ± 0.089*** **	**1.501 ± 0.070*** **	**1.515 ± 0.070*** **	**1.456 ± 0.077*** **	**1.416 ± 0.077*** **
	**BLA**	0.662 ± 0.048	0.676 ± 0.049	**0.822 ± 0.060*** **	**1.079 ± 0.064*** **	**0.976 ± 0.056*** **	**1.084 ± 0.070*** **	**1.054 ± 0.070*** **	**1.088 ± 0.067*** **	**1.082 ± 0.073*** **
**High Gamma**	**dHPC**	0.598 ± 0.034	0.611 ± 0.033	0.596 ± 0.036	**0.881 ± 0.075*** **	**0.843 ± 0.093*** **	**0.732 ± 0.049*** **	**0.801 ± 0.071*** **	**0.830 ± 0.082*** **	**0.711 ± 0.052*** **
	**iHPC**	1.011 ± 0.066	1.039 ± 0.066	1.002 ± 0.078	**1.308 ± 0.078*** **	**1.252 ± 0.070*** **	**1.255 ± 0.074*** **	**1.287 ± 0.063*** **	**1.215 ± 0.080*** **	**1.145 ± 0.078*** **
	**vHPC**	0.523 ± 0.055	0.553 ± 0.043	**0.803 ± 0.052*** **	**1.103 ± 0.089*** **	**1.145 ± 0.085*** **	**1.140 ± 0.071*** **	**1.104 ± 0.068*** **	**1.103 ± 0.059*** **	**1.131 ± 0.068*** **
	**BLA**	0.576 ± 0.053	0.583 ± 0.053	0.554 ± 0.058	**1.019 ± 0.052*** **	**0.813 ± 0.034*** **	**0.974 ± 0.054*** **	**0.906 ± 0.056*** **	**0.943 ± 0.054*** **	**0.790 ± 0.052*** **

Note: Mean ± se (bold: statistical significance in pairwise comparisons to basal period *** p< 0.001)
