# Supplementary material for: The Oscillatory Profile Induced by the Anxiogenic Drug FG-7142 in the Amygdala–Hippocampal Network Is Reversed by Infralimbic Deep Brain Stimulation: Relevance for Mood Disorders
Source: Biomedicines. 2021 Jul 6;9(7):783. doi: 10.3390/biomedicines9070783 (PMC8301458; doi:10.3390/biomedicines9070783)
Supplement: Supplementary file 1 [file biomedicines-09-00783-s001.zip › Biomedicines supplemental/SupplTable S5.pdf]

Table S5. Phase Locking Value, measured as weighted phase lag index (WPLI).

Band	Channels	Basal	Saline	FG-7142	DBS1	DBS2	DBS3	DBS4	DBS5	POST-DBS
**Slow Waves**	**dHPC-iHPC**	0.890 ± 0.024	0.901 ± 0.018	**0.260 ± 0.016*** **	**0.398 ± 0.030*** **	**0.645 ± 0.044*** **	**0.673 ± 0.042*** **	**0.669 ± 0.047*** **	**0.710 ± 0.031*** **	0.888 ± 0.019
	**dHPC-BLA**	0.869 ± 0.024	0.865 ± 0.021	**0.350 ± 0.037*** **	**0.553 ± 0.041*** **	**0.555 ± 0.036*** **	**0.659 ± 0.047*** **	**0.686 ± 0.037*** **	**0.769 ± 0.020*** **	0.825 ± 0.035
	**iHPC-BLA**	0.896 ± 0.017	0.884 ± 0.033	**0.253 ± 0.034*** **	**0.470 ± 0.031*** **	**0.459 ± 0.041*** **	**0.714 ± 0.031*** **	**0.834 ± 0.034*** **	**0.774 ± 0.032*** **	**0.861 ± 0.019†**
	**vHPC-dHPC**	0.812 ± 0.034	0.801 ± 0.039	**0.282 ± 0.033*** **	**0.419 ± 0.034*** **	**0.698 ± 0.063*** **	**0.787 ± 0.044*** **	**0.826 ± 0.042***	**0.779 ± 0.043*** **	0.869 ± 0.028
	**vHPC-iHPC**	0.807 ± 0.039	0.813 ± 0.025	**0.255 ± 0.039*** **	**0.412 ± 0.047*** **	**0.579 ± 0.051*** **	0.803 ± 0.039	0.795 ± 0.036	**0.697 ± 0.037*** **	0.845 ± 0.028
	**vHPC-BLA**	0.884 ± 0.019	0.889 ± 0.026	**0.371 ± 0.034*** **	**0.749 ± 0.041***	**0.664 ± 0.048****	**0.747 ± 0.035†**	0.911 ± 0.025	0.854 ± 0.034	0.849 ± 0.035
**Delta**	**dHPC-iHPC**	0.414 ± 0.031	0.383 ± 0.033	0.428 ± 0.033	**0.532 ± 0.045†**	**0.582 ± 0.048*** **	0.458 ± 0.046	**0.556 ± 0.045*** **	**0.720 ± 0.046*** **	**0.540 ± 0.049*** **
	**dHPC-BLA**	0.583 ± 0.038	0.583 ± 0.044	0.652 ± 0.042	0.600 ± 0.048	0.596 ± 0.043	**†0.510 ± 0.043**	0.607 ± 0.050	0.544 ± 0.054	0.497 ± 0.056
	**iHPC-BLA**	0.337 ± 0.026	0.318 ± 0.022	**0.796 ± 0.029*** **	**0.664 ± 0.046*** **	**0.484 ± 0.052*** **	0.482 ± 0.048	**0.493 ± 0.042*** **	**0.425 ± 0.032*** **	0.328 ± 0.032
	**vHPC-dHPC**	0.542 ± 0.068	0.527 ± 0.065	**0.453 ± 0.081*** **	**0.389 ± 0.080*** **	0.405 ± 0.066	**0.455 ± 0.080****	**0.411 ± 0.087****	0.532 ± 0.080	0.438 ± 0.088
	**vHPC-iHPC**	0.325 ± 0.026	0.293 ± 0.037	**0.780 ± 0.034*** **	0.483 ± 0.042	**0.604 ± 0.054****	0.345 ± 0.043	0.349 ± 0.041	0.316 ± 0.026	0.326 ± 0.022
	**vHPC-BLA**	0.367± 0.035	0.359± 0.051	**0.811± 0.041*** **	**†0.588± 0.067**	**0.761± 0.045*** **	**0.607± 0.066****	**0.705± 0.061*** **	0.497± 0.083	0.213± 0.030
**Low Theta**	**dHPC-iHPC**	0.218± 0.024	0.252± 0.026	**0.771± 0.028*** **	**0.693± 0.039*** **	**0.689± 0.043*** **	**0.652± 0.045*** **	**0.714± 0.052*** **	**0.374± 0.043*** **	**0.266± 0.031***
	**dHPC-BLA**	0.227± 0.027	0.282± 0.030	**0.767± 0.028*** **	**0.524± 0.044*** **	**0.518± 0.038*** **	0.366± 0.033	**0.328± 0.037****	0.247± 0.029	**0.259± 0.037***
	**iHPC-BLA**	0.339± 0.040	0.334± 0.044	**0.290± 0.045*** **	**0.328± 0.050****	0.415± 0.060	**0.320± 0.051*** **	0.413± 0.057	0.348± 0.039	0.424± 0.062
	**vHPC-dHPC**	0.291± 0.033	0.257± 0.033	**0.801± 0.032*** **	**0.613± 0.039****	0.519± 0.044	**0.591± 0.057***	**0.597± 0.071***	0.331± 0.035	0.321± 0.033
	**vHPC-iHPC**	0.291± 0.016	0.285± 0.044	**0.827± 0.042*** **	0.482± 0.068	0.309± 0.054	0.497± 0.061	**0.515± 0.061****	0.343± 0.049	0.296± 0.023
	**vHPC-BLA**	0.239± 0.025	0.273± 0.022	**0.788± 0.032*** **	**0.527± 0.061****	**0.475± 0.053*** **	0.435± 0.061	0.382± 0.051	0.312± 0.033	0.213± 0.024
**Beta**	**dHPC-iHPC**	0.091± 0.013	0.102± 0.012	0.097± 0.011	0.109± 0.012	**0.139± 0.017*** **	**0.117± 0.015***	0.124± 0.015	**0.135± 0.017*** **	**0.128± 0.015*** **
	**dHPC-BLA**	0.110± 0.014	0.108± 0.015	0.118± 0.013	**0.131± 0.013*** **	**0.125± 0.012†**	**0.133± 0.016*** **	**0.130± 0.015***	**0.141± 0.018*** **	**0.154± 0.014*** **
	**iHPC-BLA**	0.168± 0.012	0.164± 0.014	**0.419± 0.023*** **	**0.433± 0.027*** **	**0.343± 0.031*** **	**0.246± 0.017****	0.158± 0.019	0.169± 0.014	0.167± 0.011
	**vHPC-dHPC**	0.163± 0.018	0.135± 0.028	0.147± 0.027	**0.127± 0.022***	0.106± 0.016	0.159± 0.019	0.135± 0.024	0.138± 0.025	0.115± 0.028
	**vHPC-iHPC**	0.111± 0.017	0.109± 0.019	**0.411± 0.023*** **	**0.310± 0.029*** **	**0.291± 0.038*** **	**0.242± 0.027***	**0.353± 0.034*** **	0.097± 0.023	0.115± 0.011
	**vHPC-BLA**	0.143± 0.022	0.146± 0.015	**0.482± 0.018*** **	**0.280± 0.026*** **	**0.289± 0.041***	0.233± 0.028	0.179± 0.030	0.177± 0.025	0.142± 0.021

Note: Mean ± se (bold: statistical significance in pairwise comparisons to basal period; ***p< 0.001;, **p< 0.01, *p< 0.05; †: 0.08<p<0.05)
