# Supplementary material for: The Oscillatory Profile Induced by the Anxiogenic Drug FG-7142 in the Amygdala–Hippocampal Network Is Reversed by Infralimbic Deep Brain Stimulation: Relevance for Mood Disorders
Source: Biomedicines. 2021 Jul 6;9(7):783. doi: 10.3390/biomedicines9070783 (PMC8301458; doi:10.3390/biomedicines9070783)
Supplement: Supplementary file 1 [file biomedicines-09-00783-s001.zip › Biomedicines supplemental/SupplTable S6.pdf]

Table S6. Phase-amplitude coupling (measured as modulation index).

Bands	Region	Basal	Saline	FG-7142	DBS1	DBS2	DBS3	DBS4	DBS5	POST-DBS
Delta-Beta	dHPC	0.0012 ± 0.0001	0.0011 ± 0.0001	0.0011 ± 0.0001	**0.0028 ± 0.0002*****	**0.0022 ± 0.0002*****	**0.0022 ± 0.0002*****	**0.0022 ± 0.0002*****	**0.0021 ± 0.0002*****	**0.0024 ± 0.0002*****
	iHPC	0.0032 ± 0.0003	0.0028 ± 0.0001	**0.0076 ± 0.0009*****	**0.0041 ± 0.0003*****	**0.0044 ± 0.0004*****	0.0034 ± 0.0003	0.0031 ± 0.0003	0.0035 ± 0.0004	0.0032 ± 0.0003
	vHPC	0.0013 ± 0.0001	0.0013 ± 0.0002	**0.0035 ± 0.0003*****	**0.0037 ± 0.0004*****	**0.0025 ± 0.0001*****	**0.0020 ± 0.0002*****	**0.0027 ± 0.0002*****	**0.0021 ± 0.0002*****	**0.0016 ± 0.0002**†
	BLA	0.0028 ± 0.0002	0.0027 ± 0.0003	**0.0043 ± 0.0003*****	0.0031 ± 0.0002	0.0024 ± 0.0002	**0.0023 ± 0.0002**†	**0.0020 ± 0.0002*****	**0.0022 ± 0.0003*****	**0.0021 ± 0.0002*****
Slow Waves– Low Theta	dHPC	0.0122 ± 0.0010	0.0117 ± 0.0013	**0.0185 ± 0.0010*****	**0.0270 ± 0.0023*****	**0.0188 ± 0.0020*****	**0.0204 ± 0.0021*****	**0.0228 ± 0.0018*****	0.0123 ± 0.0015	0.0114 ± 0.0006
	iHPC	0.0231 ± 0.0012	0.0230 ± 0.0018	**0.0317 ± 0.0029*****	**0.0295 ± 0.0023*****	0.0263 ± 0.0020	0.0256 ± 0.0022	**0.0305 ± 0.0031****	**0.0194 ± 0.0012****	**0.0193 ± 0.0018***
	vHPC	0.0092 ± 0.0008	0.0084 ± 0.0009	**0.0186 ± 0.0015*****	**0.0168 ± 0.0026*****	**0.0159 ± 0.0018*****	**0.0138 ± 0.0013***	**0.0149 ± 0.0015****	0.0092 ± 0.0010	0.0104 ± 0.0008
	BLA	0.0108 ± 0.0009	0.0114 ± 0.0009	**0.0195 ± 0.0018*****	**0.0180 ± 0.0016*****	0.0137 ± 0.0022	**0.0190 ± 0.0010*****	0.0098 ± 0.0010	0.0108 ± 0.0013	0.0108 ± 0.0009
Slow Waves – Low Gamma	dHPC	0.0013 ± 0.0002	0.0012 ± 0.0002	0.0011 ± 0.0002	**0.0035 ± 0.0004*****	**0.0016 ± 0.0001***	**0.0023 ± 0.0001*****	**0.0024 ± 0.0002*****	**0.0026 ± 0.0002*****	**0.0027 ± 0.0002*****
	iHPC	0.0039 ± 0.0002	0.0038 ± 0.0003	0.0039 ± 0.0004	**0.0066 ± 0.0005*****	**0.0056 ± 0.0004*****	**0.0058 ± 0.0005*****	**0.0056 ± 0.0005*****	**0.0066 ± 0.0005*****	**0.0062 ± 0.0005*****
	vHPC	0.0014 ± 0.0001	0.0014 ± 0.0002	0.0016 ± 0.0002	**0.0034 ± 0.0003*****	**0.0044 ± 0.0004*****	**0.0032 ± 0.0003*****	**0.0026 ± 0.0004*****	**0.0028 ± 0.0004****	**0.0029 ± 0.0004*****
	BLA	0.0012 ± 0.0001	0.0012 ± 0.0001	0.0011 ± 0.0001	**0.0035 ± 0.0002*****	**0.0025 ± 0.0004*****	**0.0024 ± 0.0003*****	**0.0024 ± 0.0003*****	**0.0022 ± 0.0002*****	**0.0036 ± 0.0003*****
Slow Waves – Mid Gamma	dHPC	0.00049 ± 0.00003	0.00048 ± 0.00003	0.00049 ± 0.00004	**0.00124± 0.00010*****	**0.00084 ± 0.00007*****	**0.00082 ± 0.00006*****	**0.00089 ± 0.00009*****	**0.00084 ± 0.00007*****	**0.00084 ± 0.00006*****
	iHPC	0.00095 ± 0.00007	0.00097 ± 0.00006	0.00097 ± 0.00005	**0.00149± 0.00011*****	**0.00127 ± 0.00012*****	**0.00129 ± 0.00016*****	**0.00119 ± 0.00011****	**0.00125 ± 0.00012*****	**0.00118 ± 0.00011****
	vHPC	0.00079 ± 0.00007	0.00079 ± 0.00005	0.00076 ± 0.00006	**0.00137± 0.00016*****	**0.00110 ± 0.00011****	0.00091 ± 0.00006	**0.00107 ± 0.00012***	0.00087 ± 0.00006	**0.00117 ± 0.00006****
	BLA	0.00057 ± 0.00004	0.00061 ± 0.00005	0.00060 ± 0.00005	**0.00094± 0.00007*****	**0.00069 ± 0.00006***	**0.00085 ± 0.00006*****	**0.00083 ± 0.00006*****	**0.00086 ± 0.00005*****	**0.00083 ± 0.00004*****
Slow Waves – High Gamma	dHPC	0.00043 ± 0.00003	0.00038 ± 0.00002	0.00038 ± 0.00003	**0.00062± 0.00006*****	0.00052 ± 0.00008	0.00047 ± 0.00002	**0.00058 ± 0.00007***	**0.00057 ± 0.00004***	**0.00068 ± 0.00007*****
	iHPC	0.00068 ± 0.00007	0.00076 ± 0.00006	0.00078 ± 0.00009	**0.00137± 0.00012*****	**0.00106 ± 0.00012*****	**0.00100 ± 0.00013*****	**0.00096 ± 0.00013*****	**0.00095 ± 0.00009*****	**0.00093 ± 0.00009*****
	vHPC	0.00040 ± 0.00004	0.00041 ± 0.00005	0.00041 ± 0.00005	**0.00145± 0.00011*****	**0.00069 ± 0.00008*****	**0.00093 ± 0.00010*****	**0.00091 ± 0.00008*****	**0.00077 ± 0.00008*****	**0.00113 ± 0.00012*****
	BLA	0.00031 ± 0.00003	0.00033 ± 0.00003	0.00032 ± 0.00003	**0.00091± 0.00008*****	**0.00046 ± 0.00004*****	**0.00047 ± 0.00005****	**0.00046 ± 0.00006****	**0.00050 ± 0.00006*****	**0.00063 ± 0.00008*****
Low Theta – Low Gamma	dHPC	0.0004 ± 0.0000	0.0004 ± 0.0000	0.0004 ± 0.0000	**0.0018 ± 0.0002*****	**0.0017 ± 0.0003*****	**0.0010 ± 0.0001*****	**0.0010 ± 0.0001*****	**0.0010 ± 0.0001*****	**0.0012 ± 0.0001*****
	iHPC	0.0012 ± 0.0001	0.0012 ± 0.0001	**0.0016 ± 0.0001*****	**0.0025 ± 0.0002*****	**0.0023 ± 0.0002*****	**0.0035 ± 0.0002*****	**0.0025 ± 0.0002*****	**0.0032 ± 0.0003*****	**0.0032 ± 0.0002*****
	vHPC	0.0007 ± 0.0001	0.0007 ± 0.0002	0.0008 ± 0.0002	**0.0020 ± 0.0002*****	**0.0015 ± 0.0002*****	**0.0017 ± 0.0002*****	**0.0013 ± 0.0001****	**0.0014 ± 0.0002*****	**0.0015 ± 0.0002*****
	BLA	0.0004 ± 0.0001	0.0004 ± 0.0000	0.0004 ± 0.0000	**0.0017 ± 0.0002*****	**0.0011 ± 0.0001*****	**0.0009 ± 0.0001*****	**0.0008 ± 0.0001*****	**0.0011 ± 0.0001*****	**0.0012 ± 0.0001*****
Mid Theta – Low Gamma	dHPC	0.00027±0.00004	0.00021±0.00002	**0.00036± 0.00002*****	**0.00078± 0.00007*****	**0.00046± 0.00007*****	**0.00032±0.00002***	**0.00033±0.00003***	**0.00030±0.00002***	0.00032±0.00004
	iHPC	0.00051±0.00004	0.00048±0.00003	**0.00042±0.00005****	**0.00072±0.00005*****	0.00062±0.00007	0.00047±0.00006	**0.00041± 0.00005*****	0.00047±0.00005	**0.00032± 0.00005*****
	vHPC	0.00028±0.00004	0.00032±0.00006	0.00031±0.00005	**0.00099± 0.00010*****	**0.00096± 0.00010*****	**0.00080± 0.00012*****	**0.00067± 0.00012*****	**0.00048±0.00009***	**0.00057± 0.00008*****
	BLA	0.00021±0.00003	0.00023±0.00003	0.00023±0.00004	**0.00037± $4*10^{-5}$*****	0.00023±0.00003	**0.00028±0.00004***	0.00029±0.00004	0.00026±0.00003	0.00023±0.00003
High Theta – Low Gamma	dHPC	0.00020±0.00003	0.00020±0.00003	**0.00034± 0.00002*****	**0.00034± 0.00003*****	0.00020 ± 0.00005	0.00019 ± 0.00001	**0.00013 ± 0.00003***	**0.00011 ± 0.00001*****	0.00014 ± 0.00002
	iHPC	0.00031±0.00005	0.00028±0.00002	**0.00096± 0.00006*****	**0.00066± 0.00006*****	0.00027 ± 0.00003	0.00032 ± 0.00004	0.00031 ± 0.00005	0.00030 ± 0.00004	0.00031 ± 0.00004
	vHPC	0.00017±0.00002	0.00017±0.00002	0.00021±0.00004	**0.00095± 0.00008*****	0.00020 ± 0.00002	0.00020 ± 0.00003	0.00020 ± 0.00003	0.00022 ± 0.00004	0.00023 ± 0.00001
	BLA	0.00020±0.00003	0.00018±0.00003	0.00016±0.00003	**0.00075± 0.00007*****	**0.00014 ± 0.00002**†	0.00015 ± 0.00002	0.00019 ± 0.00002	0.00018 ± 0.00002	0.00023 ± 0.00006

Note: Mean ± se (bold: statistical significance in pairwise comparisons to basal period; ***$p < 0.001$, **$p < 0.01$; *$p < 0.05$; †: $0.08 < p < 0.05$)
