# Supplementary figures and images for: The Oscillatory Profile Induced by the Anxiogenic Drug FG-7142 in the Amygdala–Hippocampal Network Is Reversed by Infralimbic Deep Brain Stimulation: Relevance for Mood Disorders
Source: Biomedicines. 2021 Jul 6;9(7):783. doi: 10.3390/biomedicines9070783 (PMC8301458; doi:10.3390/biomedicines9070783)

# SLOW WAVES

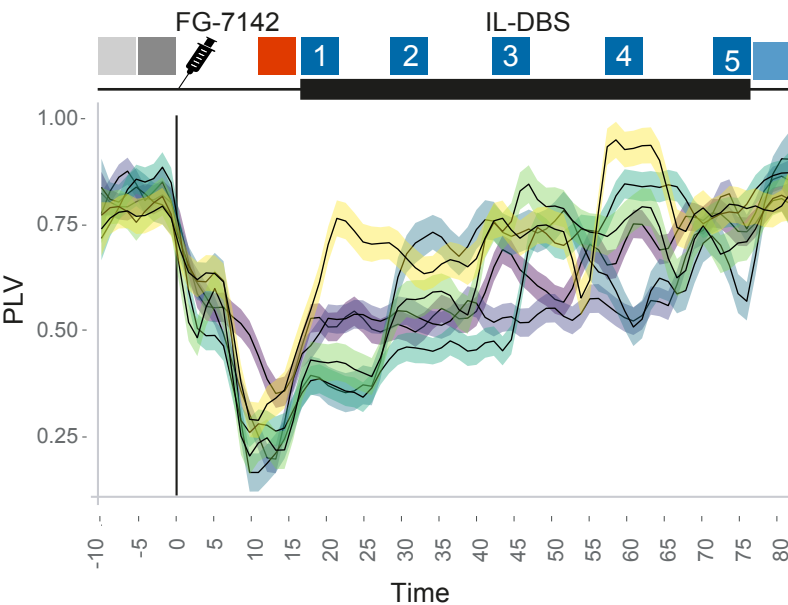

# PLV DELTA

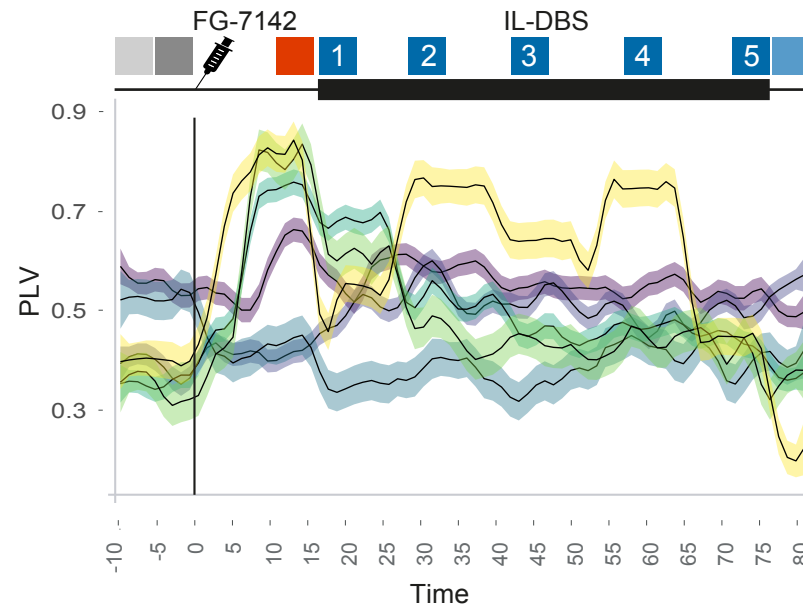

# LOW THETA

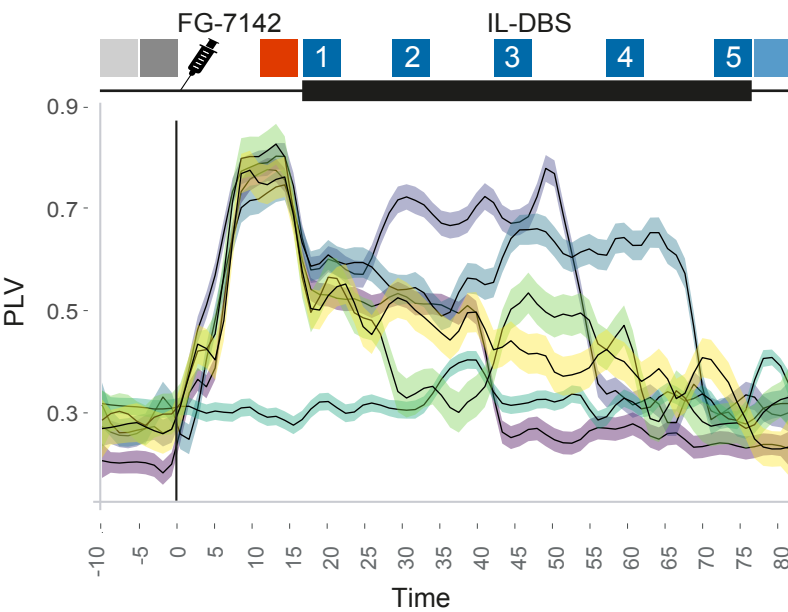

# PLV BETA

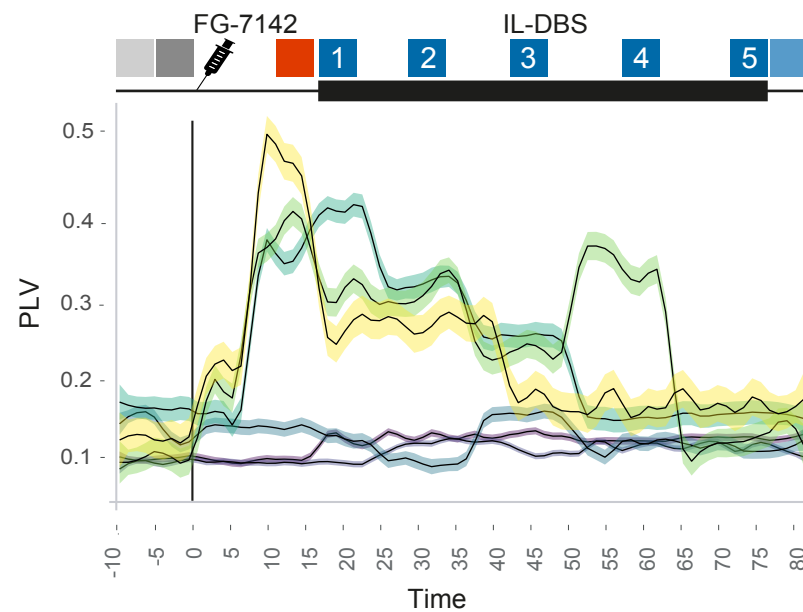

- dHPC-BLA
- dHPC-iHPC
- dHPC-vHPC
- iHPC-BLA
- iHPC-vHPC
- vHPC-BLA

Supplement: Supplementary file 1 [file biomedicines-09-00783-s001.zip › Biomedicines supplemental/Figure S1.pdf]

# Supplementary Figure S2

## dHPC

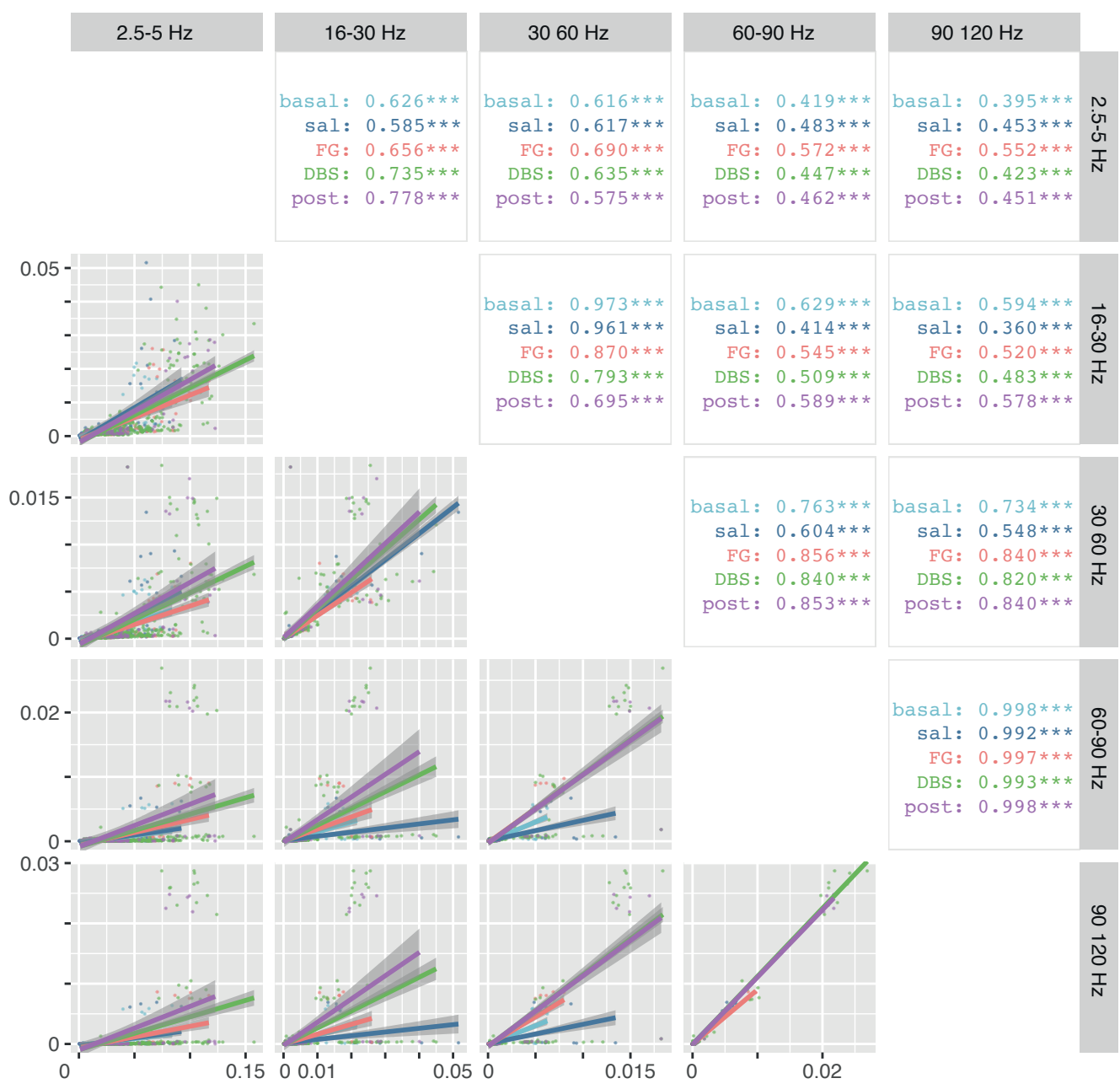

## iHPC

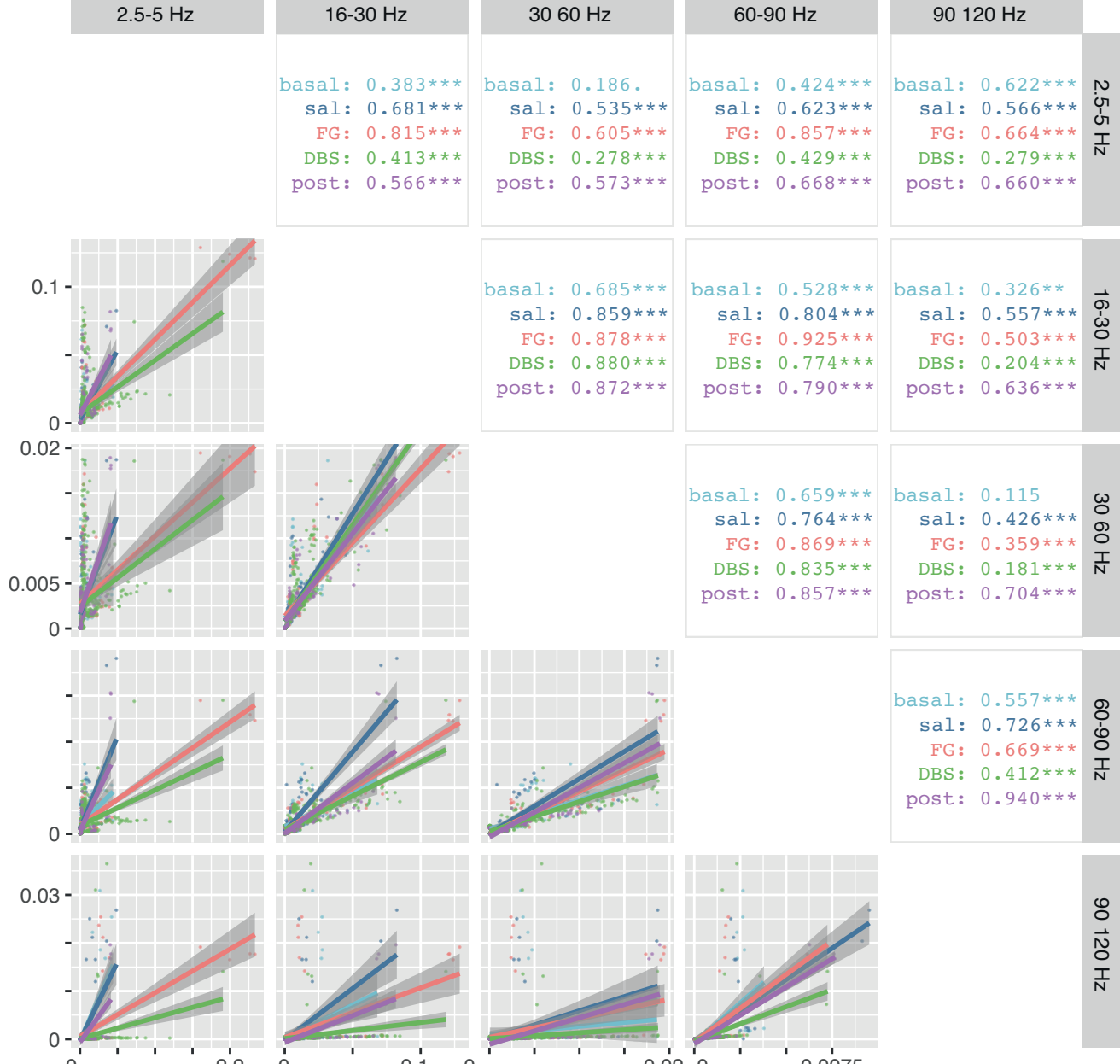

## vHPC

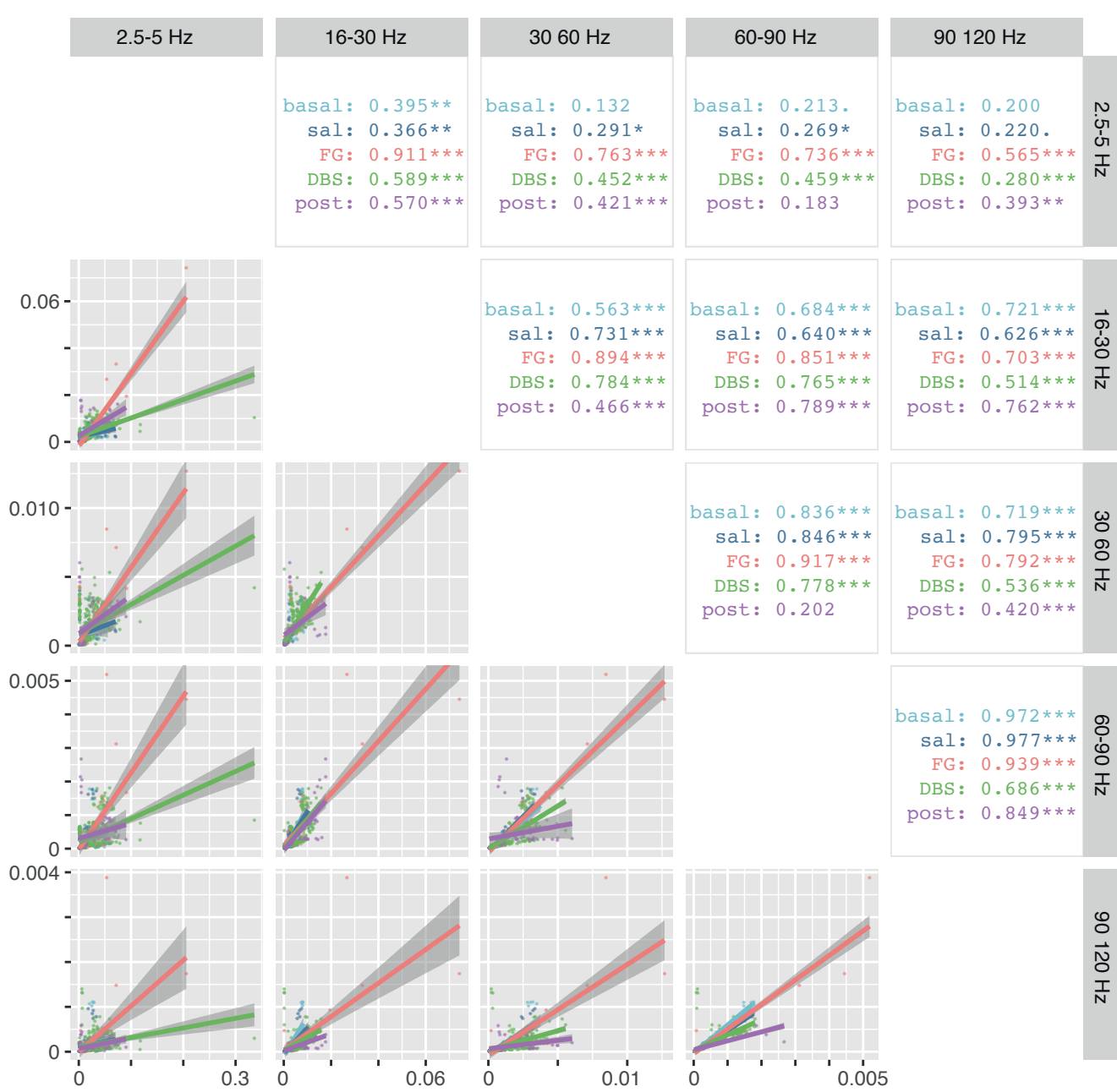

## BLA

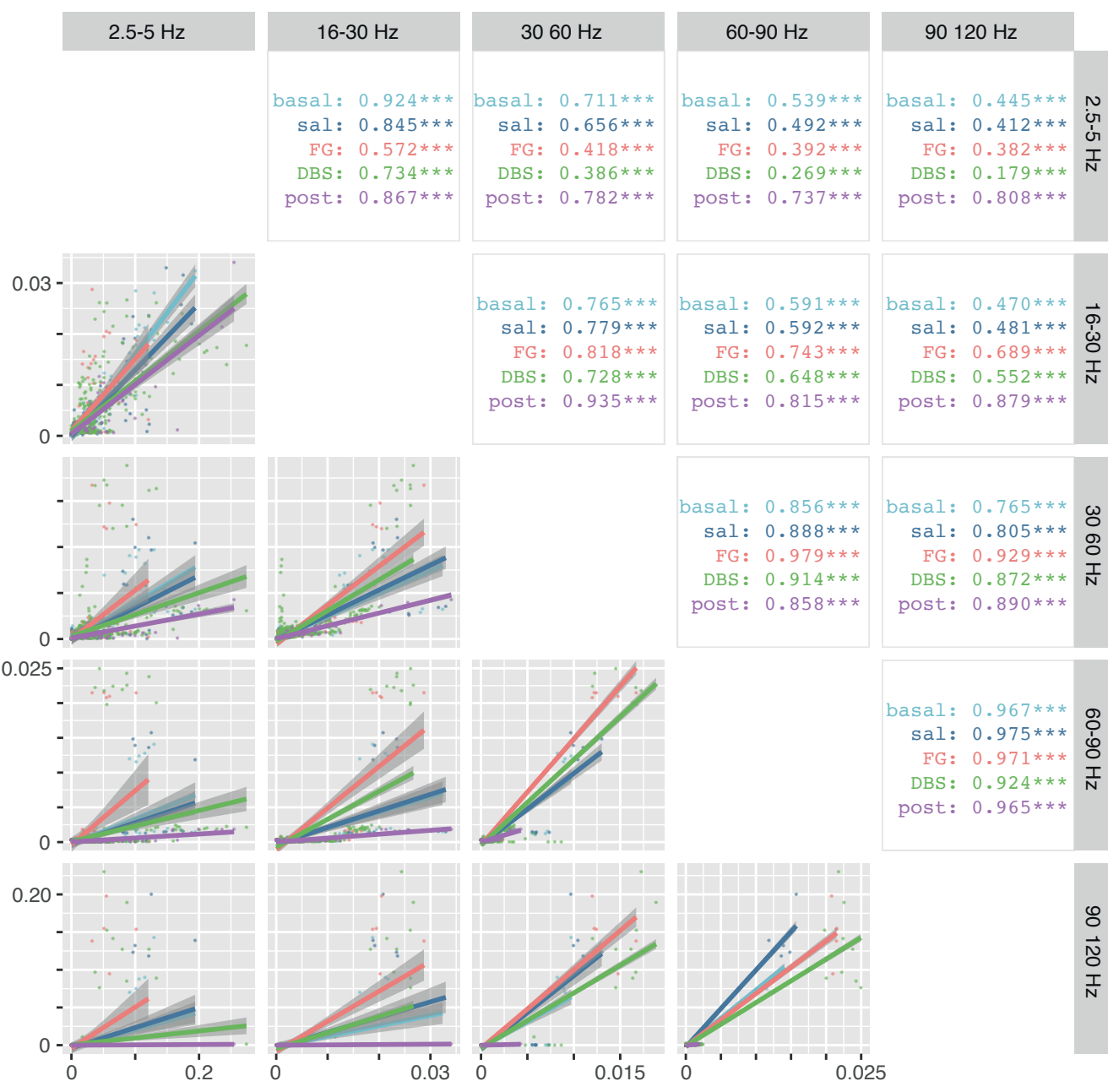

Supplement: Supplementary file 1 [file biomedicines-09-00783-s001.zip › Biomedicines supplemental/Figure S2.pdf]
